# *Chlamydia trachomatis* Cell-to-Cell Spread through Tunneling Nanotubes

**DOI:** 10.1128/spectrum.02817-22

**Published:** 2022-10-11

**Authors:** Rico Jahnke, Svea Matthiesen, Luca M. Zaeck, Stefan Finke, Michael R. Knittler

**Affiliations:** a Institute of Immunology, Friedrich-Loeffler-Institut, Federal Research Institute of Animal Health, Greifswald, Germany; b Institute of Molecular Virology and Cell Biology, Friedrich-Loeffler-Institut, Federal Research Institute of Animal Health, Greifswald, Germany; University of Guelph

**Keywords:** cell infection, chlamydia, inclusions

## Abstract

Tunneling nanotubes (TNTs) are transient cellular connections that consist of dynamic membrane protrusions. They play an important role in cell-to-cell communication and mediate the intercellular exchanges of molecules and organelles. TNTs can form between different cell types and may contribute to the spread of pathogens by serving as cytoplasmic corridors. We demonstrate that Chlamydia (*C.*) trachomatis-infected human embryonic kidney (HEK) 293 cells and other cells form TNT-like structures through which reticulate bodies (RBs) pass into uninfected cells. Observed TNTs have a life span of 1 to 5 h and contain microtubules, which are essential for chlamydial transfer. They can bridge distances of up to 50 μm between connecting neighboring cells. Consistent with the biological role for TNTs, we show that C. trachomatis spread also occurs under conditions in which the extracellular route of chlamydial entry into host cells is blocked. Based on our findings, we propose that TNTs play a critical role in the direct, cell-to-cell transmission of chlamydia.

**IMPORTANCE** Intracellular bacterial pathogens often undergo a life cycle in which they parasitize infected host cells in membranous vacuoles. Two pathways have been described by which chlamydia can exit infected host cells: lytic cell destruction or exit via extrusion formation. Whether direct, cell-to-cell contact may also play a role in the spread of infection is unknown. Tunneling nanotubes (TNTs) interconnect the cytoplasm of adjacent cells to mediate efficient communication and the exchange of material between them. We used Chlamydia trachomatis and immortalized cells to analyze whether TNTs mediate bacterial transmission from an infected donor to uninfected acceptor cells. We show that chlamydia-infected cells build TNTs through which the intracellular reticulate bodies (RBs) of the chlamydia can pass into uninfected neighboring cells. Our study contributes to the understanding of the function of TNTs in the cell-to-cell transmission of intracellular pathogens and provides new insights into the strategies by which chlamydia spreads among multicellular tissues.

## INTRODUCTION

Chlamydiae are Gram-negative, obligate intracellular bacteria that are responsible for numerous pathologies (1). The organism Chlamydia (*C.*) trachomatis (serovars D-K and L1-3) is the most common cause of bacterial sexually transmitted infections (STIs) worldwide (2). Like other chlamydial species, C. trachomatis has a biphasic developmental cycle. Outside host cells, the organisms exist as infectious elementary bodies (EBs) (1) but do not exhibit translational or metabolic activity (3, 4). Once taken up by a host cell, the EBs are sequestered into inclusions (5) and transform into reticulate bodies (RBs). Such RBs have active protein translation and metabolism, and they proliferate within the host cell (3). Multiple EBs can enter the same host cell, leading to numerous intracellular RB-containing inclusions, which can then merge through homotypic fusion into one larger vacuole (1). Chlamydial RBs multiply inside these inclusions via a unique polarized budding mechanism (6). At the end of the chlamydial developmental cycle, the RBs transform back into EBs, which are then released through host cell lysis and/or the formation of extrusions, to infect neighboring cells (1). This final step in the relationship between the host cell and the pathogen is conventionally believed to act as the main strategy promoting chlamydial spread.

The initial binding of chlamydial EBs to the host cell is a multistep process that involves electrostatic interactions with heparan sulfate-like glycosaminoglycans (GAGs) (7). Consequently, chlamydial entry is blocked by physiological concentrations (1 to 5 μg/mL) of heparin (8–10), which effectively function as “decoy receptors” (11). How chlamydia overcomes heparin-mediated inhibition *in vivo* and thereby succeeds in infecting tissues and spreading within organs is unknown. Direct cell contacts may accelerate chlamydial spread, although the underlying mechanism is unclear (12).

Tunneling nanotubes (TNTs) are a group of cellular connections consisting of long, open membrane conduits that mediate cell-to-cell communication (13, 14) in addition to performing a variety of other physiological and pathological functions. Many primary and immortalized cell types form and use these flexible, dynamic structures, which range from 25 to 1,000 nm in diameter and 10 to 100 μm in length (15–17). Their lifetimes can range from a few minutes to several hours (18), and their cytoskeletal content is heterogeneous, containing F-actin and/or microtubules (MTs) (13, 14). MTs likely serve as tracks for motor protein-dependent transport and may provide rigidity to a tube, thereby extending its half-life (19). The formation of these cytoplasmic bridges between neighboring cells allows for the efficient exchange of signals, molecules, and organelles (13, 14). However, the means by which the transfer of cytoplasmic material through TNTs is controlled *in vivo* is unknown, but it probably involves active energy-dependent processes.

TNT-mediated communication and transport might be important for normal cell function under physiological conditions. Additionally, these processes could play crucial roles in the pathogenesis of various diseases (14). Several viral and bacterial pathogens also exploit TNTs to spread from cell to cell (13, 14, 20–23) while limiting their exposure to the humoral immune system (13).

Here, we examined whether C. trachomatis can use TNTs for dissemination into neighboring cells. Indeed, we demonstrate the presence of chlamydial RBs within transient, up to 50 μm long, TNT-like structures, which form between neighboring infected and uninfected cells. Strikingly, these TNTs are maintained, even if one of the two cell partners dies at a later stage of the chlamydial infection. Such nanotubes contain MTs and motor proteins, supporting the hypothesis that C. trachomatis may actively migrate along these tracks. Importantly, this particular type of cell-to-cell transfer occurs in the presence of physiological heparin concentrations, which block the extracellular route of dissemination. Thus, our findings provide a plausible mechanism for chlamydial spread within multicellular tissues *in vivo*.

## RESULTS

### Presence of chlamydial structures within interconnecting TNTs of infected host cells.

**FIG 1:**
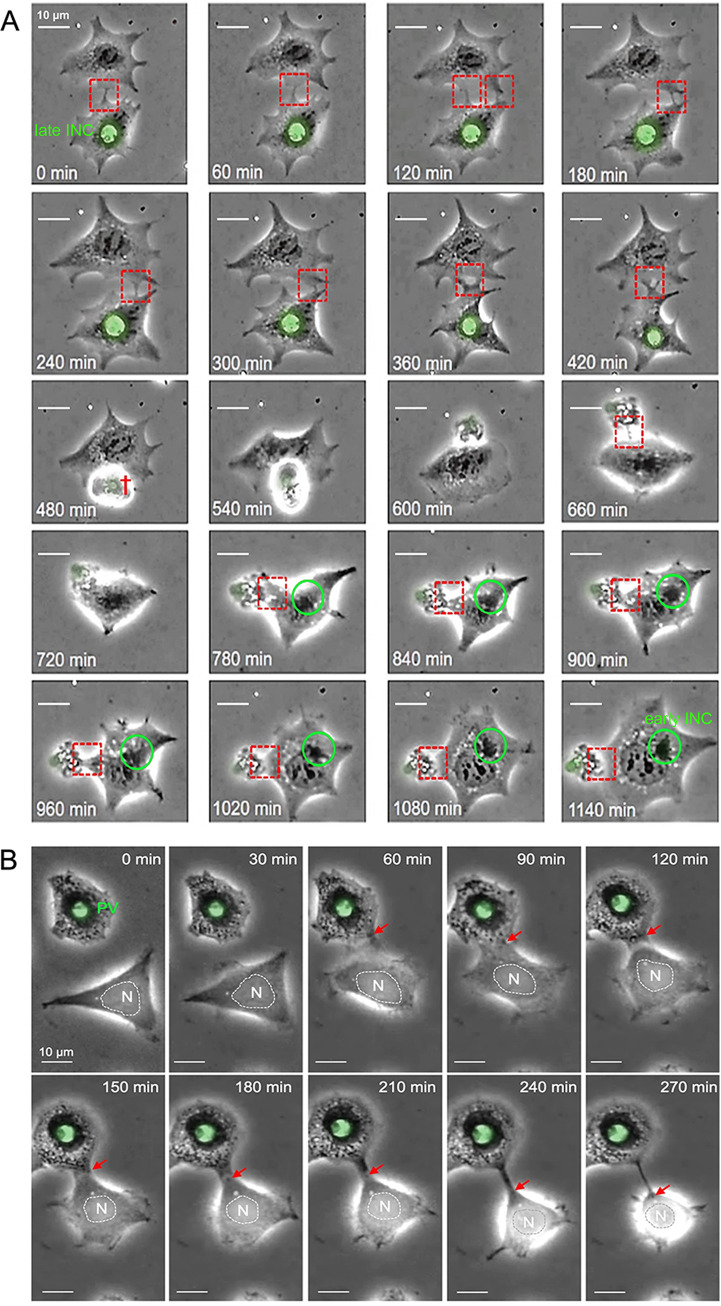
Long-term and short-term time-lapse recordings of TNT-formation between chlamydia-infected and uninfected partner cells. A total of 1×10^5^ cells were infected with green-fluorescent C. trachomatis (C. trachomatis *LGV2/434/Bu/pGFP::SW2*) (multiplicity of infection [MOI] of 3, washed 3 hpi, and cultivated in the presence of heparin), and live-cell imaging (LCI) was started at 30 hpi. (A) Long-term time-lapse recording. The movement of bacteria was followed for a duration of 1,140 min at 37°C. (B) Short-term time-lapse recording. TNT formation and bacterial movement were followed for a duration of 270 min at 37°C. Timestamps are relative to the start of the respective image series in panels A and B. The dashed red boxes and red arrows in panels A and B highlight the dynamics of TNTs formed between HEK293 partner cells. The green circles in panel A mark the formation of an early inclusion (INC). The dying partner cell in panel A is indicated by a red cross at the 480 min mark. Late INCs and nuclei (N) are labeled in panels A and B. The corresponding LCI movies of panels A and B are available upon request.

**FIG 2:**
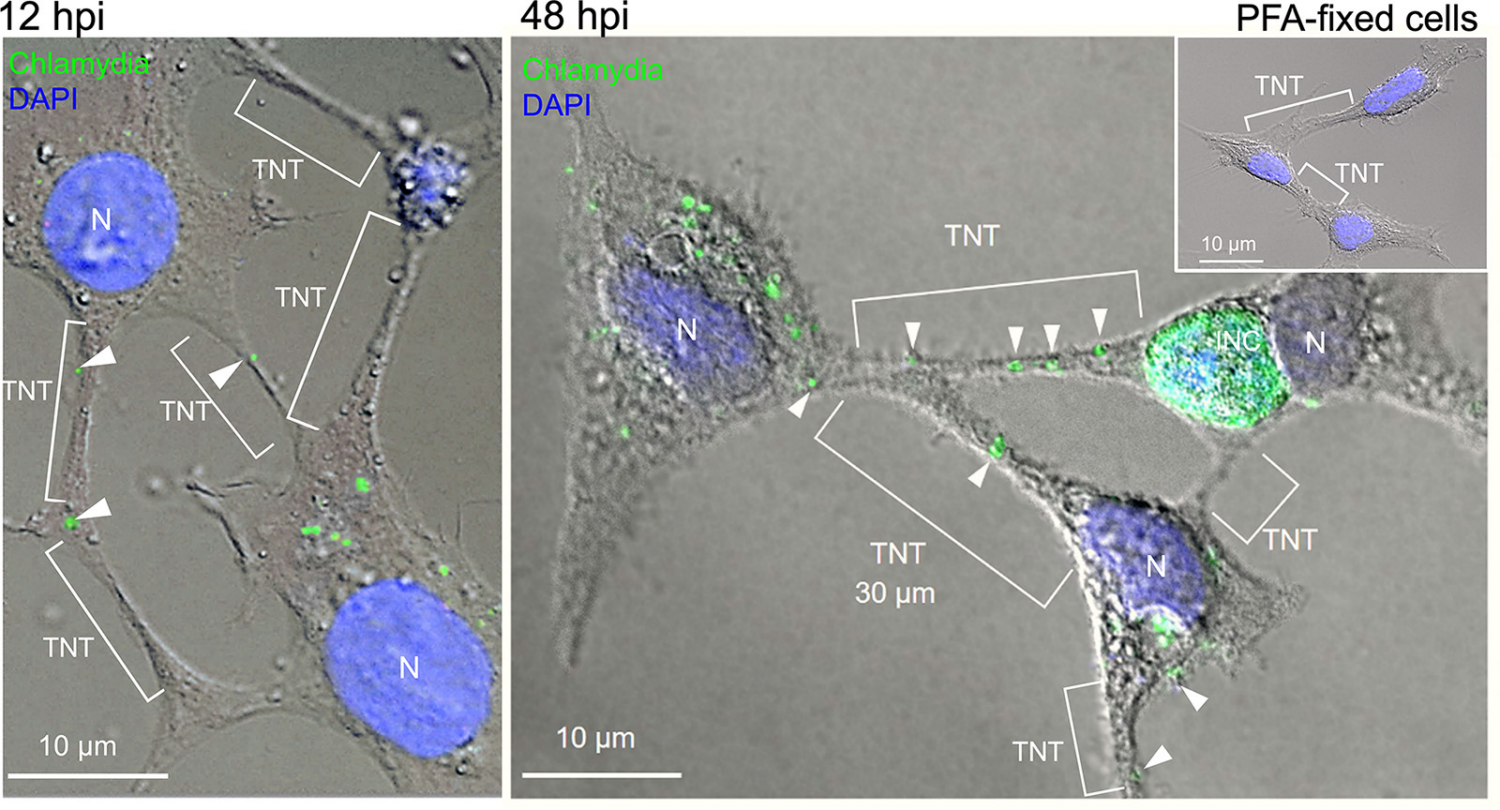
Presence of chlamydia within the cytoplasm and interconnecting TNTs of infected cells. Immunofluorescence analysis of chlamydia (green) (left, 12 hpi; right, 48 hpi) in infected PFA-fixed HEK293 cells (MOI = 3). DNA was stained via DAPI (blue). Fluorescence and corresponding phase-contrast images were taken and overlaid to visualize the presence of interconnecting TNTs. White arrowheads indicate chlamydial structures found in the context of TNTs in the two overlay images. The small image inset in the upper right corner shows interconnecting TNTs in noninfected control HEK293 cells. TNTs, INCs, and nuclei (N) are indicated in the overlay image.

**FIG 3:**
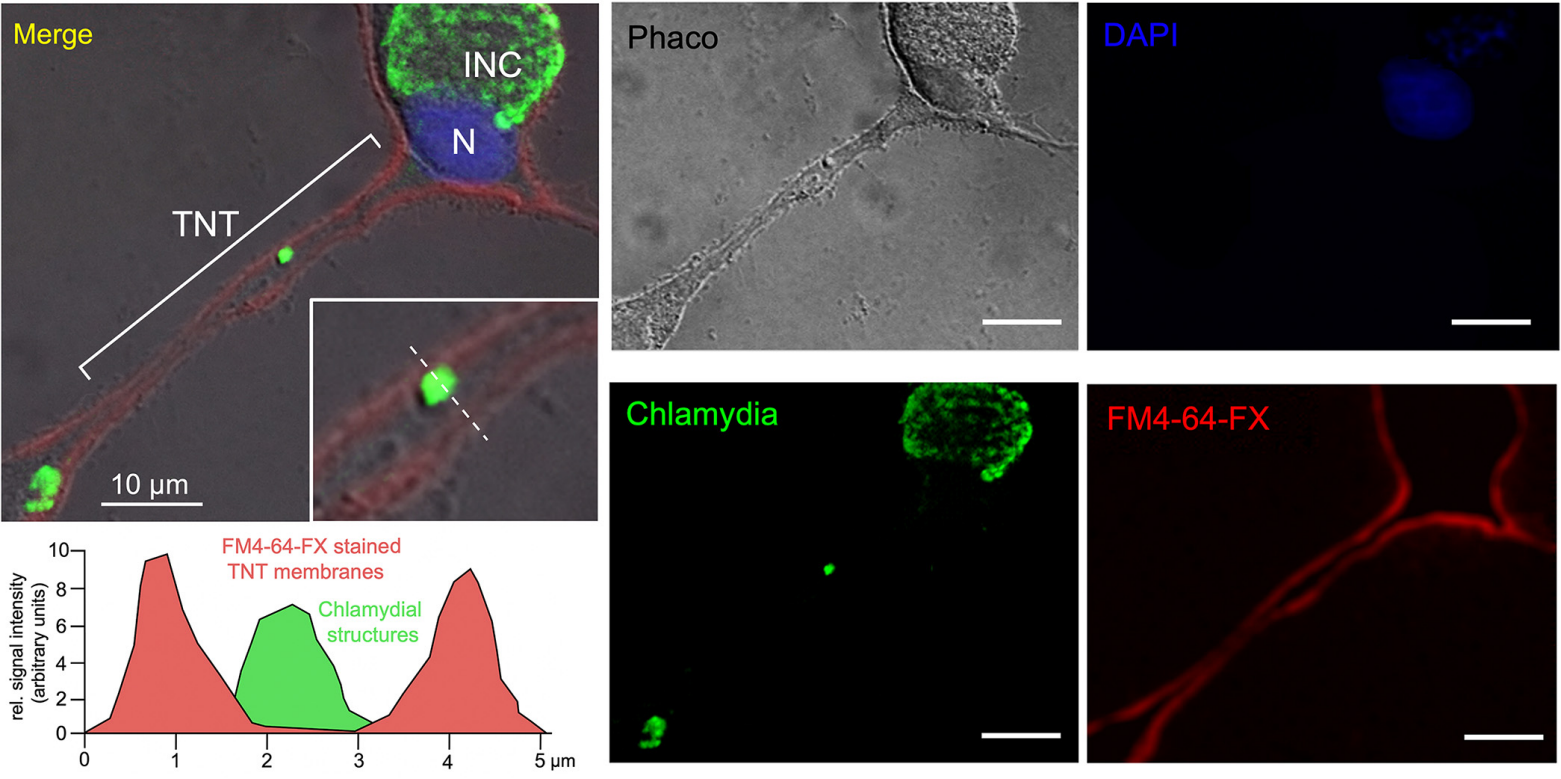
Chlamydial structures are located within rather than outside the TNTs. Immunofluorescence analysis via Apotome optical sectioning of chlamydia (green) in infected PFA-fixed HEK293 cells (48 hpi, MOI = 3). DNA was stained via DAPI (blue). The dye FM4-64-FX (Thermo Fisher Scientific) was used to selectively stain the plasma membranes of infected cells with red fluorescence. Fluorescence and corresponding phase-contrast Z-stack images of a central optical plane were taken and overlaid to visualize the cellular border, the red-stained plasma membrane, and the localization of the bacteria. TNT, INC, and nuclei (N) are indicated in the overlay image. The image in the upper left corner shows a magnification of the TNT bearing the stained chlamydial structure. The fluorescence intensity along a TNT cross-section was measured for the individual fluorophores (ImageJ), and the obtained profiles were overlaid and colored.

To address a possible role for tunneling nanotubes (TNTs) in chlamydial biology, we performed live-cell imaging (LCI) on human embryonic kidney (HEK) 293 cells infected with GFP-expressing C. trachomatis LGV2. HEK293 cells have been described as nonphagocytic and capable of forming TNTs (24–26). Our LCI experiments revealed abundant connections, frequently between infected and uninfected partners (Fig. 1; note that GFP expression is not visible earlier than 24 hpi, indicating that the fluorophore selectively labels late inclusions [27]). The connections were transient and dynamic, with an average lifetime of 1 to 5 h. They were maintained even when one of the two cells died at a later stage of infection (Fig. 1A, 480 min onward, red cross). Strikingly, while the cellular bridge was still in place, *de novo* chlamydial inclusions (with condensed perinuclear structures) appeared in the previously uninfected partner cell (Fig. 1). This was surprising because the HEK293 cells were grown in a heparin-containing medium after the initial infection, which has been demonstrated to sustainably block the extracellular route of bacterial entry (10). Indeed, our own studies (depicted in Fig. S1) show that a pretreatment with physiological concentrations of heparin (5 μg/mL) completely inhibits cell infection by extracellular C. trachomatis, whereas the posttreatment of already infected cells prevents any secondary infection by chlamydia from the outside. Thus, the presence of heparin seems ideally suited for studying the direct, cell-to-cell transmission of chlamydia by TNTs without introducing an interfering influence of extracellular bacteria. Since HEK293 cells are nonphagocytic (24–26), it is unlikely that infections result from the uptake of bacteria-containing debris via phagocytosis. In fact, the dying cell harboring the original infection often remained attached to its intact partner (e.g., at 540 min during LCI), but it was not engulfed by it, whereas the new inclusions appeared relatively late at 900 to 1,140 min (Fig. 1A, green circle). TNTs between infected and uninfected HEK293 cells are formed through cell dislodgement (the pulling apart of formed membrane connections after cell-to-cell contact) (Fig. 1B, red arrow), which is a well-known mechanism for the establishment of transient cell-to-cell connections (28). Interestingly, we observed small, dynamic, light-dense structures within the interconnecting TNTs (Fig. 1B, red arrows, 150 to 270 min). However, bacteria-associated fluorescence was not visible within the membrane conduits (Fig. 1B), presumably due to the physical detection and resolution limits of the LCI experiments. Consequently, we investigated whether C. trachomatis can enter and migrate through cellular nanotubes using higher resolution immunofluorescence microscopy. Indeed, we observed bacteria not only in the cell body but also within the TNTs between connected cells at both early (12 hpi) and late (48 hpi) time points (Fig. 2). However, the number of chlamydia-positive TNTs was significantly increased at 48 hpi. Corresponding control experiments showed that TNTs were also detectable in noninfected HEK293 cells (Fig. 2), which is expected in this system (29–31). Thus, chlamydia may be passive beneficiaries of the establishment of TNTs rather than active drivers of their formation in HEK293 cells. The observed TNTs were fixable with mild paraformaldehyde treatment (≤2% PFA) (Fig. 2) and appeared to be highly variable in shape and length. Their observed diameters reached 1 to 5 μm, while their bridged distances ranged from 0.1 to 50 μm (Fig. 2). These properties are typical for MT-containing TNTs (32). Further investigation of the multiplicity of infection (MOI) and the time dependence of the presence of chlamydia in the TNTs during the infection cycle revealed that for various standard MOIs (0.5 and 1), the bacterial structures became increasingly visible in the membrane conduits between 12 hpi and 48 hpi but not at 6 hpi (Fig. S2). Their pronounced appearance in the TNTs of infected cells occurred mainly at 48 hpi. Moreover, the observed TNT localization of chlamydia does not seem to be limited to the LGV2 strain, as it was also observed for the C. trachomatis strain Serovar D (Fig. S3). To further confirm that chlamydial structures are located inside TNTs, additional immunofluorescence experiments with Z-section analyses were performed. Specifically, the outer cell membrane and interconnecting TNTs were labeled with FM4-64-FX prior to mild PFA fixation and the costaining of intracellular chlamydia (Fig. 3). Indeed, chlamydial structures were found between the FM4-64-FX-stained TNT-lining membranes, demonstrating that bacteria reside inside the TNT lumen. Consistent with this, we also observed that membrane permeabilization is necessary for the immunofluorescence detection of TNT-localized chlamydia (Fig. S4).

### Structural and functional characteristics of chlamydia-containing TNTs between infected host cells.

**FIG 4:**
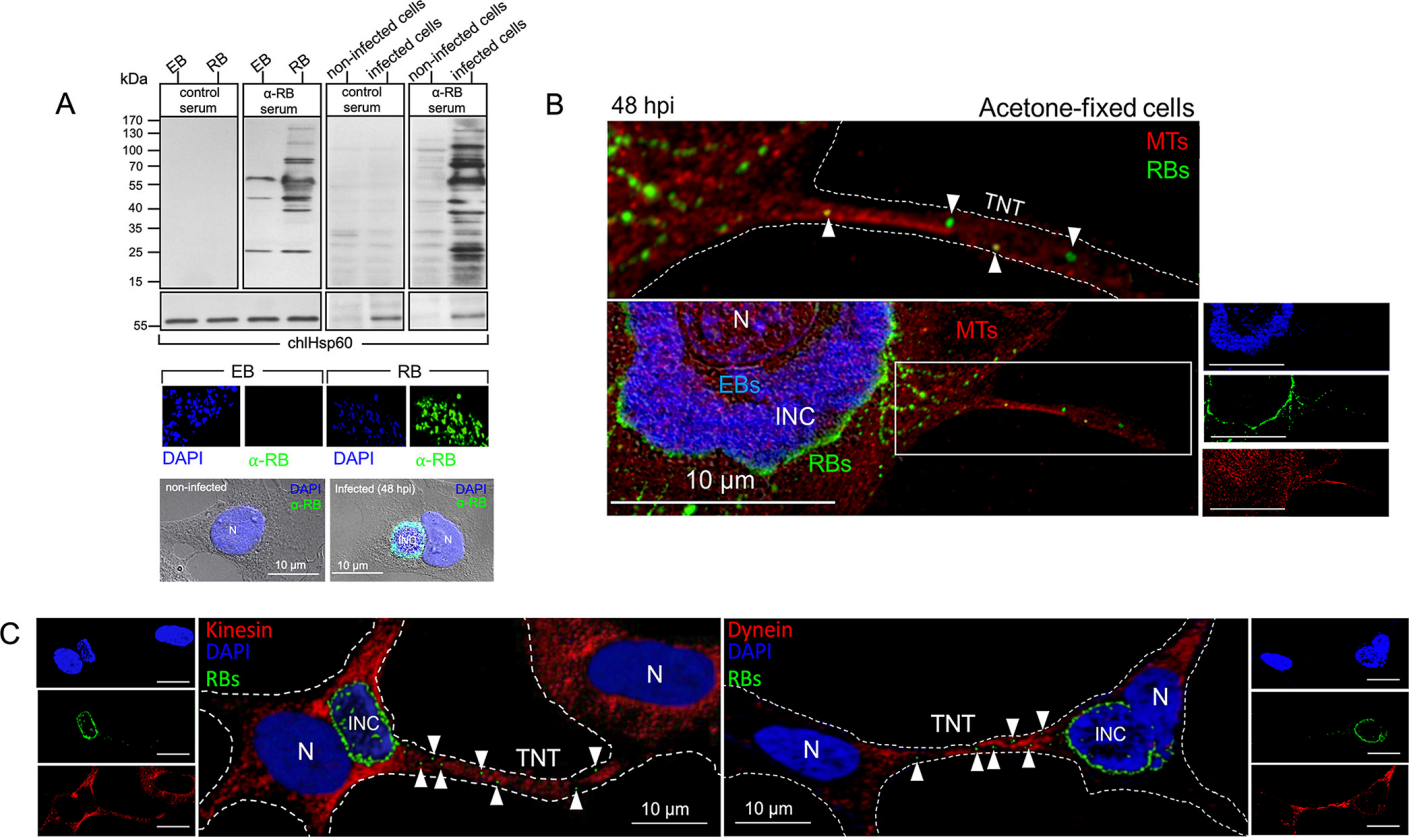
Chlamydial structures present within MT-bearing TNTs consist mainly of RBs. (A) Characterization of an RB-specific mouse antiserum. C57BL/6 mice were immunized with noninfectious chlamydial RBs. Vaccinated and control sera were used in Western blots to analyze RB/EB recognition for purified bacteria fractions and lysate extracts from noninfected and infected HEK293 cells (upper panel). Anti-mouse pan-IgG-HRP was used as a secondary antibody. The blots were additionally probed for chlHSP60 to detect both RBs and EBs. Purified EBs and RBs (middle panel), as well as noninfected and infected HEK293 cells (lower panel), were further stained in immunofluorescence with DAPI and anti-RB serum. Anti-mouse-IgG-Alexa 488 (green) was used as a secondary antibody. The RBs were stained with antiserum and, to some extent, by DAPI, whereas the EBs displayed a pronounced DAPI staining but were not recognized by the antiserum. The cellular analysis revealed specific immunostaining only for the inclusions (INCs) of infected cells (nuclei [N] are indicated). (B) HEK293 cells were infected (MOI = 5, 48 hpi), fixed with 80% acetone, and stained with the predominantly RB-recognizing anti-chlamydial serum (green). DNA was stained via DAPI (blue) and MTs with the anti-β-tubulin antibody (red). TNTs, INCs, and nuclei (N) are indicated. The corresponding image magnification above shows the presence of MTs and chlamydia within a TNT. (C) HEK293 cells were infected (MOI 5, 48 hpi), fixed with 80% acetone, and stained with anti-kinesin (red, left panel) and anti-dynein antibodies (red, right panel). Chlamydia was stained with RB-recognizing anti-chlamydial serum (green), and DNA was stained via DAPI (blue) in both panels. TNTs, INCs, and nuclei (N) are indicated. White arrowheads indicate chlamydial structures found in TNTs. For panels B and C, it should be noted that due to the use of the dehydrating acetone fixative, the cellular and bacterial structures significantly lose their original sizes after fixation.

**FIG 5:**
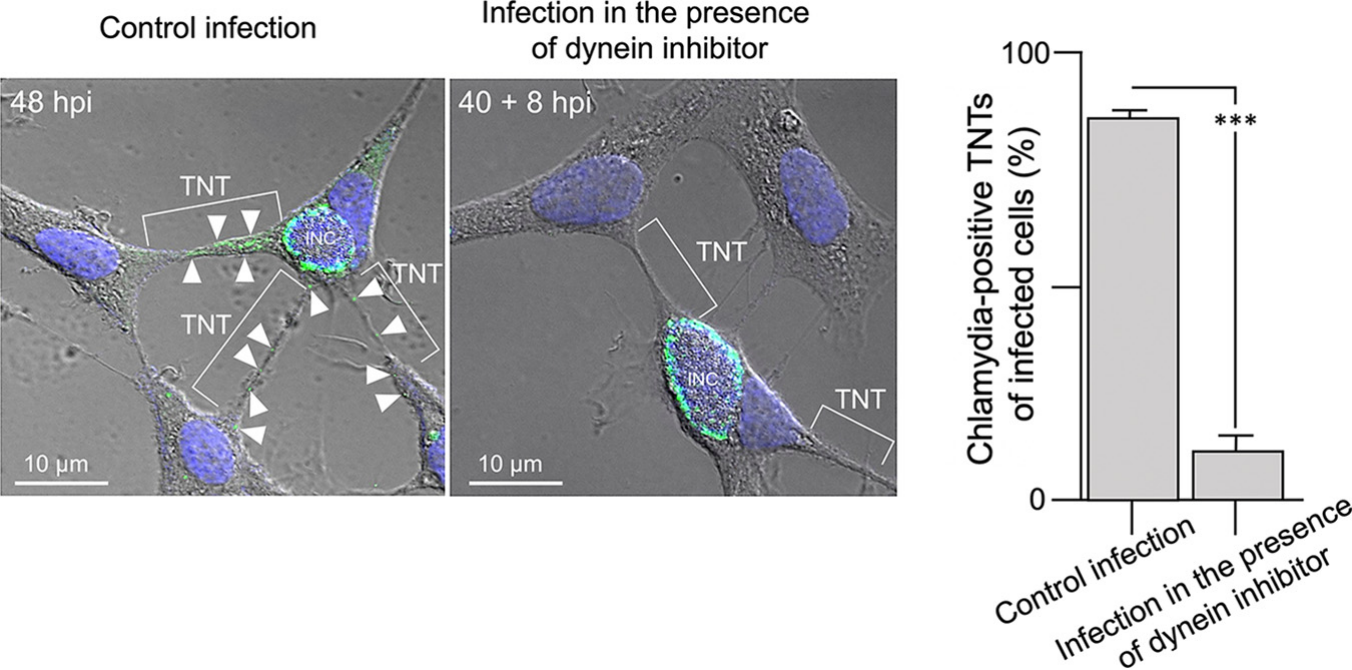
The appearance of chlamydial structures in TNTs depends on dynein function. Immunofluorescence analysis of chlamydia (green) (48 hpi) in infected PFA-fixed HEK293 cells (MOI = 3). To analyze the influence of dynein inhibition on the presence of chlamydia in TNTs, the inhibitor Dynarrestin (Merck) was added to infected cells (40 hpi) and allowed to act for a period of 8 h. The fluorescence and corresponding phase-contrast images were taken and overlaid to visualize the presence of interconnecting TNTs. DNA was stained via DAPI (blue). White arrowheads indicate chlamydial structures found in the context of visible TNTs in the two overlay images. Two representative immunofluorescence images are shown on the left (untreated control and inhibitor-treated). For each treatment condition, 3 × 100 cells connected by multiple membrane conduits were evaluated for the respective number (%) of chlamydia-positive TNTs of infected cells. The respective histogram plot is depicted on the right (***, *P* < 0.001, versus inhibitor-treated samples; *n* = 3; mean ± standard deviation [SD]).

C. trachomatis inclusions are known to exploit the host cell MT network and motor machinery for intracellular migration (33). This raised the question of whether MTs, motor proteins, and the intracellular bacterial form of chlamydia (RBs) colocalize in TNTs. To address this, C. trachomatis-infected HEK293 cells were fixed with acetone, which preserves the integrity of cellular MTs, before host cell β-tubulin and chlamydial RBs (Fig. 4A shows the corresponding characterization of the anti-RB antiserum that preferentially recognizes RBs over EBs) were specifically costained (Fig. 4B). These experiments demonstrated the presence of both MTs and RBs along the TNTs (Fig. 4B). Additionally, MT-associated motor proteins, such as kinesin and dynein, were located inside the nanotubes (Fig. 4C). Hence, cytosolic cargo likely moves between connected partner cells on MT tracks through TNTs. Interestingly, chlamydial EBs, which were visualized here exclusively by extranuclear DAPI staining (Fig. 4A), were never found within cellular connections in these experiments (Fig. 4B). Thus, our results suggest that intracellular RBs selectively use the TNT structures to migrate from cell to cell and that this migration may occur along MTs. In addition to MTs, F-actin was also found to be a structural component of chlamydia-containing TNTs between two interconnected HEK293 cells (Fig. S5). Since dynein appears to play a crucial role in chlamydial transport (34), the influence of its inhibition on chlamydial TNT presence was analyzed. Therefore, the specific inhibitor Dynarrestin was added to infected cells (40 hpi) and allowed to act for a short period of 8 h, as extended dynein blockage leads to the severe impairment of the chlamydial inclusion formation and development cycle (35). The number of chlamydia-positive TNTs was significantly reduced (or disappeared completely) in the presence of the inhibitor compared with that observed in infected control cells, suggesting that dynein indeed plays an important role in the TNT-mediated transport of chlamydia (Fig. 5).

### TNT-mediated transport of chlamydia involves a directional cytoplasmic exchange between infected donor and noninfected acceptor cells.

To further investigate the cell-to-cell spread of C. trachomatis, a two-color fluorescent cell culture system was developed. To this end, we generated two fluorescent HEK293 cell lines, a chlamydial donor cell line expressing cytoplasmic RFP as TNT cargo (16, 36, 37), and an acceptor cell line expressing GFP-tagged β-tubulin (β-tub-GFP) (Fig. S6 shows a corresponding cell staining comparison of acetone-fixed and PFA-fixed HEK293 transfectants). β-tubulin inserts into the polymerized MTs of interconnecting TNTs (38–40) but does not represent a soluble cargo for cell-to-cell transfer (31, 32, 38, 41, 42).

**FIG 6:**
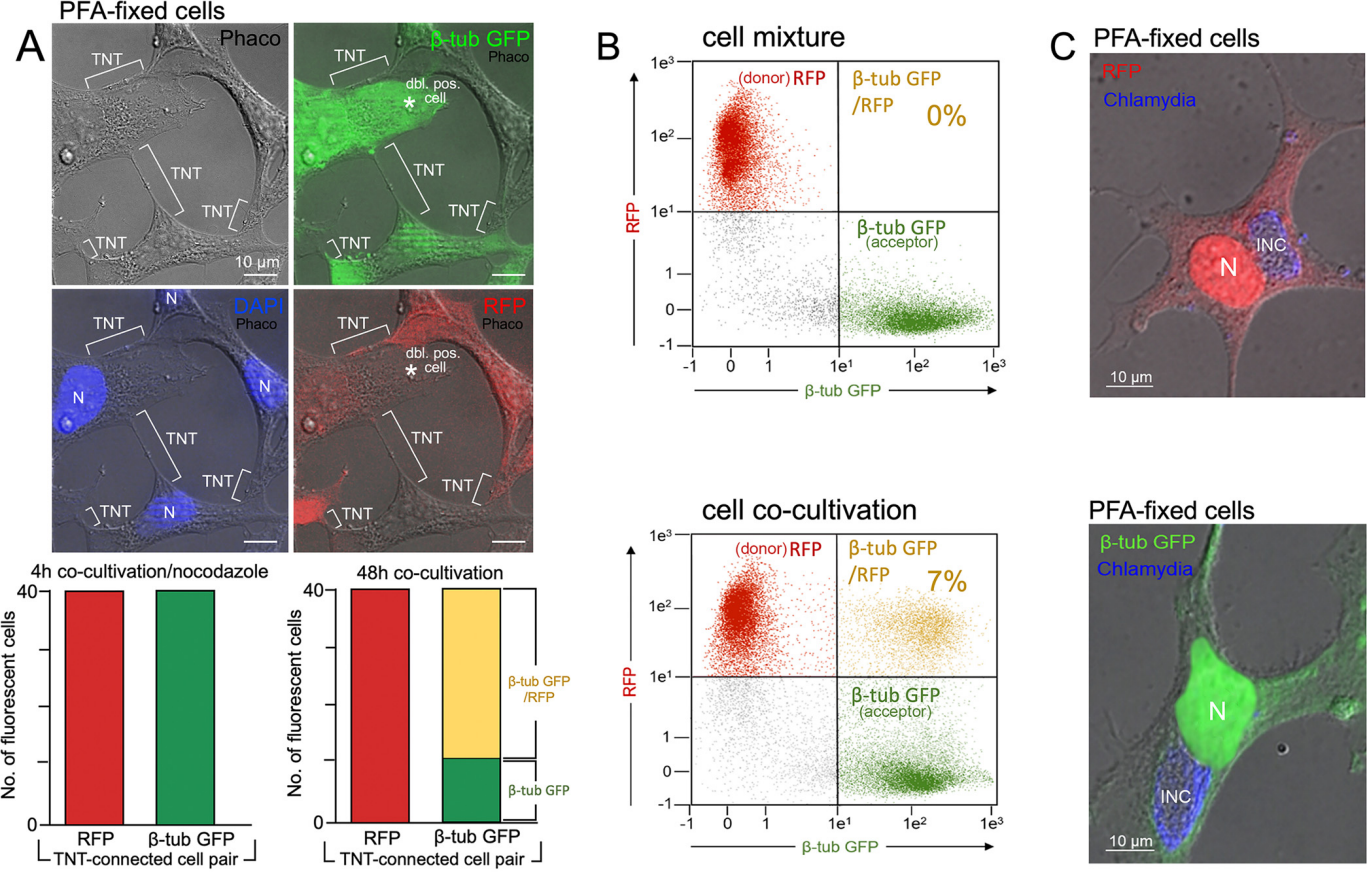
Cocultivation and chlamydial infection of stable RFP- and β-tub-GFP-HEK293 transfectants. (A) HEK293 cells were stably transfected with plasmids for RFP- or β-tub-GFP expression. Clonal selection of the two different HEK293 transfectants was performed via serial dilution in 96-well plates, followed by FACS sorting. A 1:1 coculturing of the two transfectants (48 h cultivation) revealed a detectable TNT-formation between red- fluorescent and green-fluorescent HEK293-transfectants. TNTs and nuclei (N) are indicated (upper panel). 40 red/green-cell pairs connected by TNTs were evaluated for their single and/or double fluorescence for RFP and GFP visible in the two partner cells (lower right panel). The corresponding control experiment was performed with cells that were cultivated separately and then mixed 1:1 for 4 h in the presence of the MT-destabilizer nocodazole (Merck, 50 ng/mL) (lower left panel). (B) Flow cytometry was used to analyze the directed transmission of cytoplasm from red donor cells (with freely diffusible cytoplasmic RFP) to green acceptor cells (with cell-structuring β-tub-GFP). For the cytoplasm transfer experiment, red and green HEK293 cells were either mixed briefly (upper panel) or cocultured for 48 h (lower panel) and then analyzed by flow cytometry to determine the quantity of costained cells containing both β-tub-GFP and TNT-transmitted RFP (yellow). (C) Immunofluorescence analysis of chlamydia (blue, Alexa 405-coupled secondary antibody) in separately infected RFP- and β-tub-GFP HEK293 cells (48 hpi, MOI = 3). The fluorescence and corresponding phase-contrast images were taken and then overlaid. INCs and nuclei are indicated.

Coculturing donor and acceptor cells resulted in TNT formation between them (Fig. 6A, upper left and right panels), confirming that these structures do not originate from cell division. Donor and acceptor pairings were seen for up to 15% of the cocultured cells. Moreover, as expected, in the control cocultures (4 h cocultivation), in which the MTs were additionally disrupted using nocodazole, the acceptor cells maintained their respective initial fluorescence without showing evidence of fluorophore exchange (Fig. 6A, lower left panel). In striking contrast, an extensive transfer of RFP fluorescence from donor to acceptor cells in TNT-connected cell pairs was seen in the uninhibited long-term cocultures (48 h cocultivation) (Fig. 6A, lower right panel; Fig. 6B, compare flow cytometry between the upper and lower panels). This was reflected in a drastic decrease in the number of exclusively green-fluorescent cells in analyzed donor/acceptor pairs alongside a corresponding increase in the number of double fluorescent cells (Fig. 6A, lower right panel). The population of RFP single-positive cells in the respective donor/acceptor pairs remained unchanged (Fig. 6A, lower right panel).

**FIG 7:**
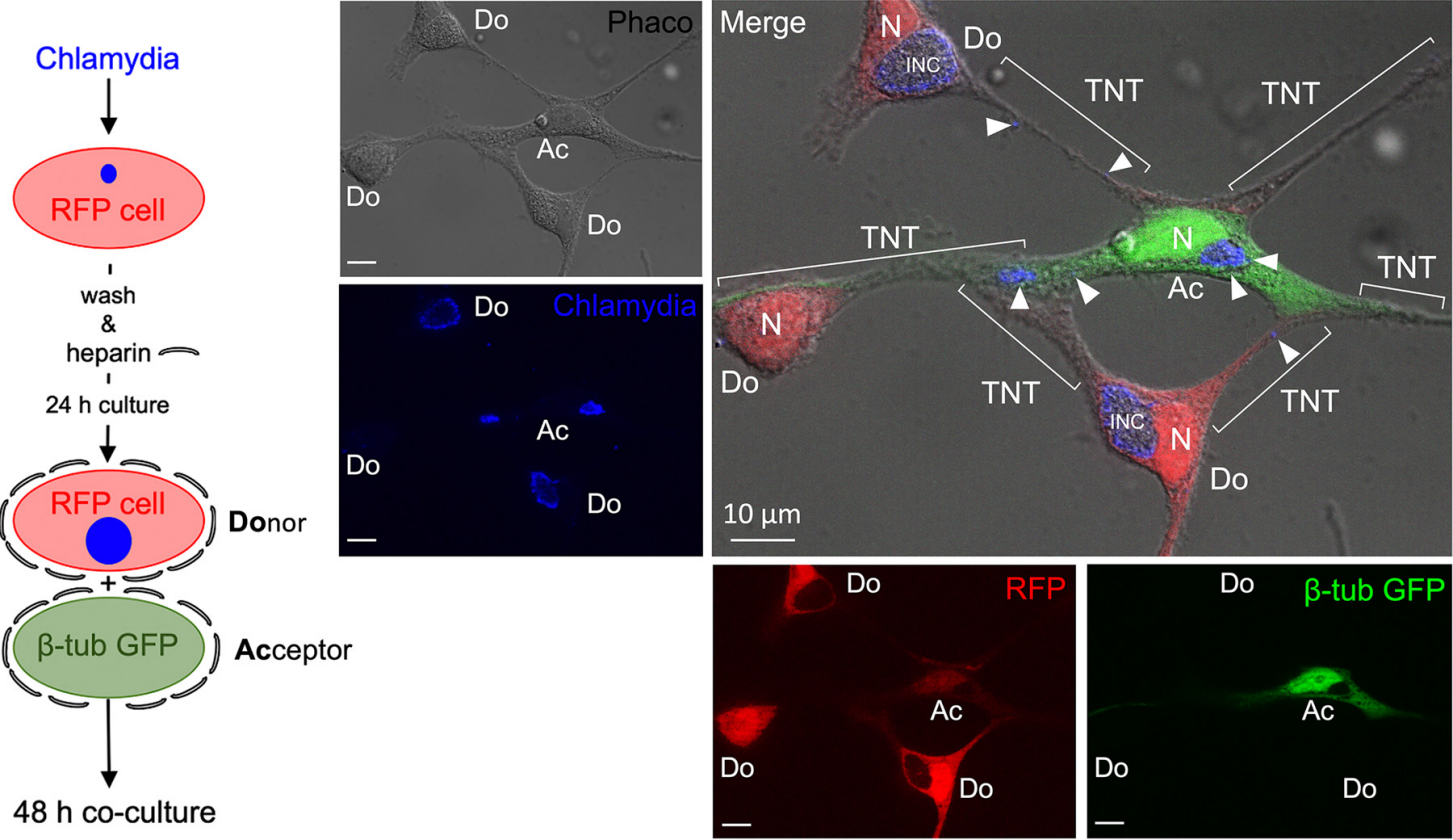
The TNT-mediated directed transmission of cytosolic material is accompanied by the cell-to-cell transfer of intracellular chlamydia. Immunofluorescence analysis of chlamydia (blue, Alexa 405-coupled secondary antibody) in infected RFP-donor HEK293 cells (24 hpi, MOI = 3) cocultured with noninfected β-tub-GFP-acceptor HEK293 cells (48 hpi) in the presence of heparin (5 μg/mL). A corresponding schematic diagram of the experimental procedure is shown on the left. Fluorescence and corresponding phase-contrast images were taken and then overlaid. Donors (Do), acceptors (Ac), inclusions (INC), nuclei (N), chlamydial structures (white arrows), and TNTs are indicated.

Next, we performed microscopic donor/acceptor experiments in the context of chlamydial infections. To this end, we first confirmed that RFP-donor and β-tub-GFP-acceptor cells showed comparable inclusion formation after infection with C. trachomatis (48 hpi) (Fig. 6C). In a subsequent experiment, donor cells were infected with C. trachomatis (MOI = 5) before they were cocultured in the presence of heparin with uninfected acceptor cells (Fig. 7). These experiments visualized RFP-positive (β-tub-GFP-negative) donor cells harboring large chlamydial inclusions (Fig. 7, labeled “Do”) TNT-linked to β-tub-GFP/RFP double-positive acceptor cells (post-RFP transfer from donor) (Fig. 7, labeled “Ac”). Strikingly, these acceptor cells acquired numerous small inclusions (white arrows/arrowheads) in addition to RFP (Fig. 7), apparently representing daughter infections originating from the connected donor cells. TNTs formed by the interconnected donor and acceptor cells are frequently attached in an antiparallel manner (Fig. S7), but the transfer of chlamydia was almost exclusively restricted to TNTs from originating acceptor cells. This points further toward the directional migration of the organisms along MTs, driven by the motor protein dynein (Fig. 4 and 5), which transports cargo from the distal plus-end to the perinuclear minus-end of MTs (43).

**FIG 8:**
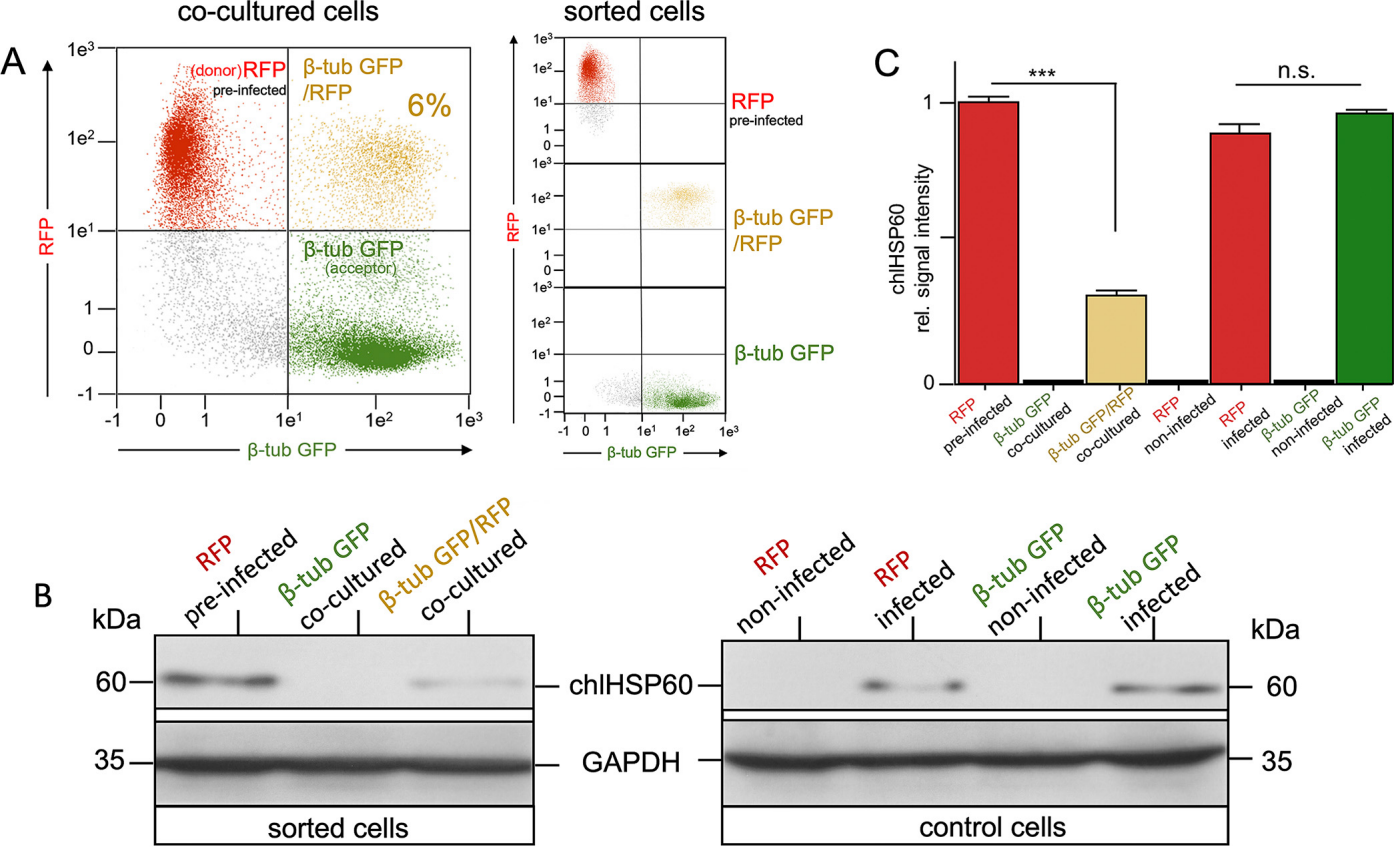
Exclusively β-tub-GFP-acceptor cells with ingested foreign cytoplasm show the acquisition of chlamydial infection from RFP-donor cells in the presence of heparin. (A) Chlamydia-infected RFP-donor HEK293 cells (MOI = 5) cocultured in the presence of heparin (5 μg/mL) with noninfected β-tub-GFP-acceptor HEK293 cells were analyzed via flow cytometry (left panel) and then separated using a FACSAria (BD) into red, green, and double-positive HEK293 cell populations. The separated cell populations were analyzed for single (red and green) and double fluorescence (red/green) using flow cytometry (right panel). (B) Sorted RFP-donor (red), β-tub-GFP-acceptor cells (green), and β-tub-GFP/RFP double-positive HEK293 cells (yellow), were washed twice with ice-cold PBS, counted, and then lysed in detergent-containing lysis buffer (10^4^ cells/10 μL). For the control experiments, lysates of infected and noninfected RFP- and β-tub-GFP-HEK293 cells were used. Finally, all cell lysates were separated via SDS-PAGE and then analyzed via Western blotting probed for chlHSP60 and GAPDH (loading control). (C) After a densitometric analysis of the Western blots, the HSP60 normalized for GAPDH was plotted. The signal obtained for preinfected RFP HEK293 cells was set to 1 (n.s., not significant; ***, *P* < 0.001, versus untreated samples; *n* = 3; mean ± SD).

Finally, to show that the TNT-mediated transmission of chlamydia is indeed coupled to the cytoplasmic exchange of interconnected cells, preinfected RFP-donor cells (Fig. 8A and B) were mixed with uninfected β-tub-GFP-acceptor cells to initially allow for chlamydial transfer. Subsequently, RFP single-positive, GFP single-positive, and β-tub-GFP/RFP double-positive cells were sorted via fluorescence-activated cell sorting (FACS) (Fig. 8A) before they were lysed and probed via Western blotting for the presence of chlamydial material using chlamydial HSP60 (chlHSP60) as a proxy (Fig. 8B and C). Exclusively green cells with no evidence of receipt of cytoplasmic RFP, presumably because they had not formed a connection with a donor cell, did not contain chlHSP60. In contrast, chlHSP60 was abundantly detected in post-RFP transfer β-tub-GFP/RFP double-positive cells, confirming that the presence of chlamydial material was correlated with the receipt of RFP by the acceptor. These observations fit the suggested scenario in which intracellular chlamydiae migrate into neighboring cells through cytoplasm-connecting TNTs.

## DISCUSSION

Two dissemination strategies for chlamydia are well-characterized in the literature (44): (i) the expulsion of EBs through host cell lysis and (ii) the extrusion of bacteria while maintaining host cell integrity. In the case of extrusions, a vacuolar structure pinches off from the cell surface, which contains a layer of cytoplasm sandwiched between the outer plasma membrane-derived lipid bilayer and the inner inclusion-derived membrane, the latter of which encases the bacteria (45, 46). The outer membrane frequently displays phosphatidylserine, which facilitates the uptake of the organisms by professional phagocytes (47). Notably, the extrusion-mediated mechanism is independent of the MT-machinery (48), and we demonstrate that the MT-motor dynein is critical for chlamydial presence in and transfer through TNTs (Fig. 5 and 7A). Thus, TNT-driven, chlamydial cell-to-cell spread appears to be functionally and mechanistically distinct from the extrusion pathway described by others.

MTs are known to serve as tracks along which TNT-associated cargo, including organelles like mitochondria, is transported and exchanged between connected cells (49, 50). Dynein and kinesin, both well-characterized microtubule motor proteins, are abundant within chlamydia-containing nanotubes (Fig. 4C). Our observation that predominantly the TNTs of acceptor cells harbor chlamydial structures (Fig. S7) points to their retrograde transport via dynein toward the perinuclear microtubule organizing center (MTOC) of the recipient cells. In line with this scenario, intracellular chlamydiae have long been known to exploit dynein for their migration along MTs (34), likely utilizing the chlamydial factor CT850 (an early to late expressed inclusion membrane protein of RBs [51]), which directly binds to the motor (51).

It has been demonstrated that the passive diffusion of material through TNTs in HEK293 cells would be inadequate for efficient TNT transmission between cells (52). Thus, one can imagine that an active mechanism transports vacuole-packed chlamydial structures and vacuolar organelles. Analogous to the dynein-docking of MT-transported C. trachomatis, the movement of mitochondria within the lumens of TNTs relies on a mitochondrial motor-binding protein (Miro1) (49), which is known to bind MT motor proteins and enable organelle-transport along MTs (50).

**FIG 9:**
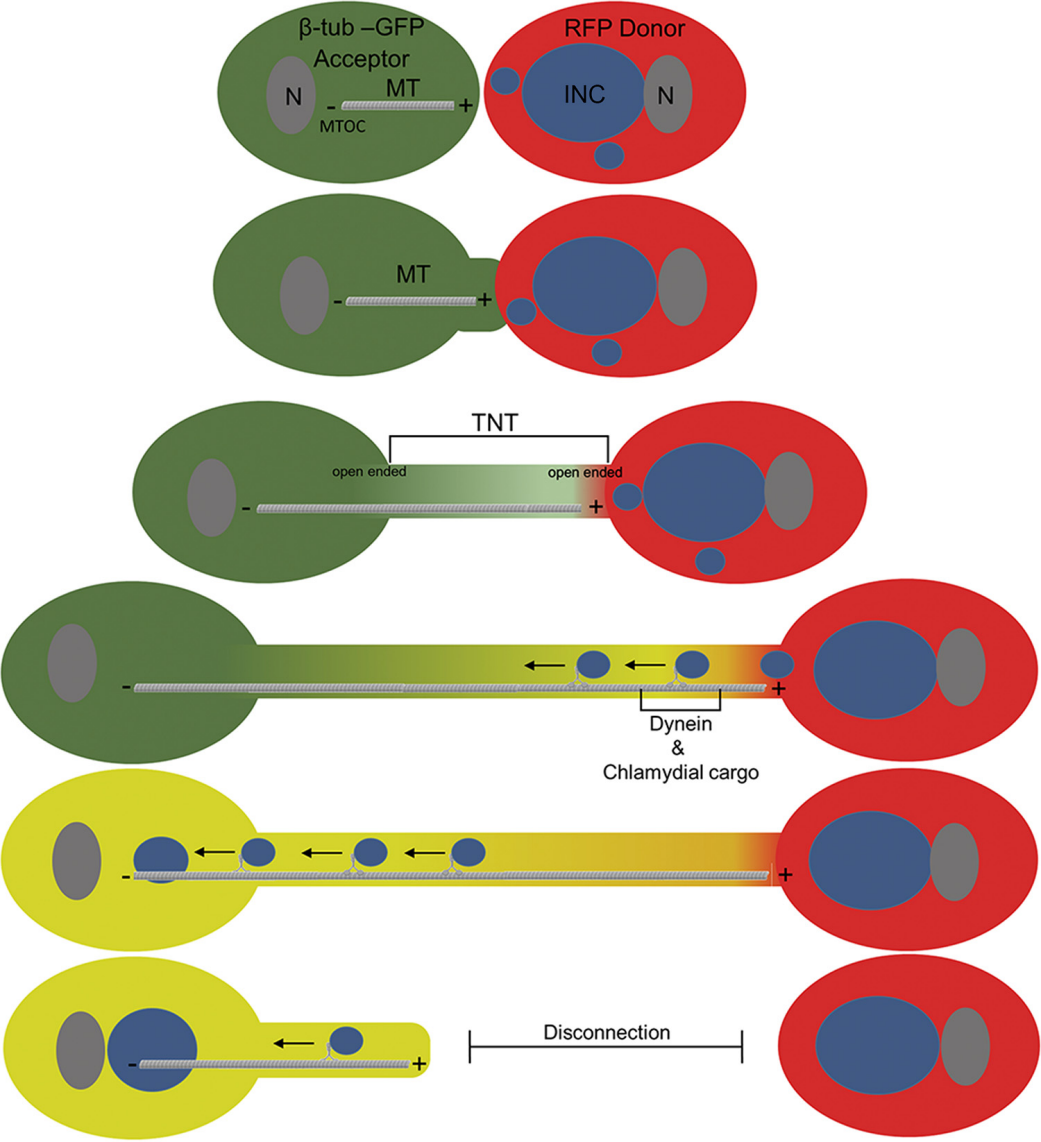
Proposed working model for the TNT-mediated transfer of chlamydia from donor to acceptor cells. MT-containing TNTs are formed via a mechanism termed “cell dislodgment” (28). In this TNT-forming process, two adjacent cell partners are initially in direct plasma membrane contact. Then, they separate from each other and become attached via a membrane conduit, which leads to an open-ended TNT and allows for cytoplasmic continuity between interconnected partner cells (a chlamydia-infected red donor cell and a noninfected green acceptor cell). The formed TNTs are bridges (representing an intercellular long-distance exchange conduit) formed by both partners with opposing plus/minus orientations of the MTs. However, for simplicity, only the TNT-formation of the noninfected acceptor cell is shown. This connection contains MTs whose minus-ends are located in the centrosome/MTOC of the acceptor cell and whose plus-ends are located in the anterior TNT tip, facing toward the infected donor cell. This MT-orientation allows for the transport of chlamydial structures into the acceptor cell body via dynein, which migrates to the “MT-terminal” centrosome/MTOC. Since the TNT structures are observed as transiently formed membrane corridors with a maximum lifetime of 5 h, it can be assumed that they subsequently dissolve and reform again elsewhere.

Based on our results, we propose a working model (Fig. 9) for TNT-mediated chlamydial transmission from infected donors to uninfected acceptors. We assume that both cell partners can form TNTs according to the hypothesis of “cell dislodgement” (28), which was also proposed and can be seen in our data from the HEK293 cells (53) (Fig. 1). In this scenario, the cell membranes of two or more partner cells fuse when they come into contact. The subsequent migration of the involved cells in opposite directions pulls out a TNT (carrying open ends on both sides). The dimensions and properties of the TNTs observed in this work (Fig. 3 and 7) correspond to those of MT-TNTs (31). For visual simplicity, only TNTs formed by acceptor cells are shown in our model (Fig. 9). Their minus-ends are in the centrosome/MTOC region of the acceptor cell, while their plus-ends are within the anterior end of the TNTs, on the side of the infected donor cells. After the bacterial transfer from the donor's cytoplasm to the TNT formed by the acceptor, the transport of the chlamydial structures via dynein follows the plus/minus MT-orientation toward the perinuclear centrosome/MTOC-region in the acceptor cell body. Since TNT structures are transient connections, it can be assumed that they are dissolved again after a certain time, during which they allow chlamydial transfer between the two partner cells.

Reactive oxygen species (ROS) are effective inducers of TNTs (40), and ROS production is transiently induced in C. trachomatis-infected cells (54). Thus, it is possible that under certain conditions, the bacteria actively promote these host reactions to induce TNT-formation and drive their spread into neighboring cells. Moreover, early apoptotic cells establish TNTs with surrounding cells to receive support by being supplied with mitochondria, which promotes survival (31, 40, 55). This could lead to the TNT-based spread of bacteria into unsuspecting host cells that rush in to help infected cells that are in distress. Further, there is evidence of an association between genital C. trachomatis strains and the development of cervical cancer (56, 57), whose degenerate cells are characterized by the presence of TNTs (58, 59). As TNTs are involved in multiple aspects of cancer development, from tumorigenesis to therapy resistance (60), investigating whether chlamydia has an impact on TNT formation and function in infected malignant tissues is an important future goal.

It has been shown that at the end of the developmental cycle, chlamydia causes the death of host cells, which is mainly driven by increased oxidative stress and displays certain mechanistic and morphological features of apoptosis (61–64). Most interestingly, neither the chlamydia-induced cell death nor the stress/apoptosis-based formation of TNTs depends on the activation of caspase 3 (31, 62, 65). Thus, one could imagine that the cell death of chlamydia-infected cells acts as a kind of final push for TNT-formation to transfer bacteria to neighboring target cells before the dying, infected host cell stops all of its physiological processes. Indeed, in cultures of chlamydia-infected mouse embryonic fibroblasts (MEFs) deficient in proapoptotic BAX, the spread of chlamydial infection to formerly uninfected cells appeared to have been impeded (44). Consistently, in BAX-deficient mice, infections were cleared faster (66). Based on these findings, it was suggested that the host cell death at the end of the chlamydial development cycle provides a more efficient and silent mode of spread (66). In line with this, our studies provide evidence that TNT-structures persist for an extended period during the cell death of the infected host cell (Fig. 1). Accordingly, chlamydia can probably be transferred from donor cell to acceptor cells until the TNT interactions are eventually resolved by the surviving acceptor cells. Further intensive studies are needed to clarify the consequences of the cell death of chlamydia-infected cells for the formation of TNTs and for the efficiency of chlamydial dissemination.

We hypothesize that TNT-mediated chlamydial dissemination may play a critical role in infections *in vivo*, as physiological heparin concentrations in serum, tissues, and organs (8, 67) may limit the classical extracellular route of infection (8, 10, 68). Additionally, this would protect the bacteria from the humoral immune response. In line with such a scenario, cellular Th1 immunity is mainly responsible for protection against C. trachomatis, and chlamydial infections are often characterized by inefficient antibody responses (69). Specifically, high titers of chlamydia-specific antibodies do not correlate with a resolution of infection in humans (70), and B cell-free mice do not show a markedly altered course of primary genital infection with C. muridarum (71). Interestingly, we observed similar cell-to-cell transfer characteristics for various chlamydial strains, including C. psittaci and C. abortus, in various host cell lines (e.g., HeLa, MN-R, and BGM) (Fig. S8), indicating that TNT-mediated transfer represents a conserved strategy across the phylum. C. trachomatis, C. abortus, and C. psittaci have distinct tropisms for specific organs (eyes, reproductive tract, and lung) (72–74), for which intercellular communication via TNTs has been described (75–77).

Previous work (78, 79) has provided evidence that the production of secondary inclusions from primary inclusions in a single cell and/or dividing daughter cells appears to occur through the active involvement of chlamydial IncA, a SNARE-like protein, which can assemble into multimeric structures. IncA is found within fibers (also termed IncA-loaded fibers) that appear as chains of vesicles between primary and secondary inclusions. Characteristically, both types of inclusions and the fiber-like interconnections between them contain RBs, and this is concordant with our observations (Fig. 4). Thus, it will be interesting to see in future studies whether IncA could also be involved in the TNT-mediated transmission of chlamydia to neighboring cells. This would potentially shed light on whether dividing daughter cells and TNT-connected cell partners use a similar mechanism for chlamydial transfer.

The involvement of TNT-based supercellularity (19) in the cell-to-cell transmission of chlamydia could have a significant impact on the nature of infections as well as on the corresponding immune responses by bypassing the developmental RB/EB switch and the need for extracellular EBs. Future work is necessary to determine the significance of this pathway *in vivo* and to reveal whether it can be pharmacologically targeted.

## MATERIALS AND METHODS

### Cell lines and cell culture.

HEK293 (ATCC no. CRL 1573) and HeLa cells (ATCC no. CCL-2) were cultivated at 37°C and 7.5% CO_2_ in Iscove's modified Dulbecco's medium (IMDM) (Thermo Fisher Scientific) containing 10% fetal bovine serum (Biochrom) and penicillin/streptomycin (Invitrogen). RFP (pTagRFP-N, Evrogen) and β-tub-GFP-constructs (pEGFP-Tub, BD Biosciences) were transfected into HEK293 cells via the DOTAP lipofection reagent (Roche Applied Science). After selection with Geneticin (G418, 0.5 mg/mL, Thermo Fisher Scientific) for 4 to 6 weeks, stable transfectants of HEK293 were subcloned, sorted, and screened via flow cytometry for RFP and β-tub-GFP expression. For the chlamydial infection experiments, the HEK293 cells and all of the generated transfectants were cultured in the presence of 5 μg/mL heparin without any antibiotics, including G418. Immortalized epithelial cells from newborn mice (MN-R cells) were obtained from the Collection of Cell Lines in Veterinary Medicine (CCLV) of the Friedrich-Loeffler-Institut (CCLV-RIE number 282). The epithelial African green monkey kidney cell line BGM was obtained from the National Reference Laboratory for Chlamydiosis of the Friedrich-Loeffler-Institut (CCLV-RIE number 136).

### Antibodies.

Antibodies against chlamydia (C. trachomatis, C. psittaci, and C. abortus), chlHSP60 (MAb A57-B9), cellular β-tubulin, and GAPDH, were obtained from Acris, Sigma-Aldrich, and Abcam. In addition, the fluorescein isothiocyanate (FITC)-conjugated mouse anti-chlamydia MAb (part of the IMAGEN Chlamydia Kit) was purchased from Oxoid. All of the secondary and isotype-control antibodies were purchased from Dianova and BioLegend.

### Preparation of anti-RB antiserum.

8-week-old C57BL/6 mice were immunized with noninfectious chlamydial RBs isolated/purified via discontinuous density gradient ultracentrifugation using Visipaque (1 mL each of 8%, 15%, 30%, and 12 mL 36%, 8 mL 40%, 5 mL 47% Visipaque) (Nycomed) (80). An EB/RB mixture containing 1× 10^9^ inclusion forming units (IFUs) served as the starting material for chlamydial purification. After ultracentrifugation (50,000 × *g*, 50 min, 4°C), the EBs were located in the boundary between the 40% and 47% Visipaque, while the RBs were found in the layer with 36% Visipaque (81). The two chlamydial fractions were checked for purity via electron microscopy (81). For vaccination, mice were immunized intraperitoneally (i.p.) with 200 μL of a purified RB/PBS suspension. Two further immunizations were given on days 14 and 28. In the control mice (littermates), the three immunization steps were carried out in parallel with sterile PBS. Seven days after the final immunization (day 35), the RB-vaccinated and control mice were sacrificed for complete blood collection. All animal procedures were approved by the local District Government (State Office for Agriculture, Food Safety, and Fishery in Mecklenburg-Western Pomerania, LALFF M-V) and were carried out according to the guidelines of the German law for the protection of animal life (LALLF M-V registration number: 7221.3-2-042/17, FLI no.: FLI 28/17).

### Western blotting.

Cells were lysed on ice in RIPA buffer (150 mM NaCl, 50 mM Tris-HCl, 1% NP-40, 0.25% Na-deoxycholate, and cOmplete protease inhibitor [Roche], 50 mM NaF) with 4 M urea. Postnuclear supernatants were analyzed via Western blot as previously described (81). Bands were visualized with enhanced chemiluminescence substrate (Sigma). Fluorographs were quantified using the GelEval 1.32 software (FrogDance Software).

### Chlamydia.

C. trachomatis serovar LGV2 strain 434/BU (BSL 2) (82) (kindly provided by Thomas Rudel, University of Würzburg, Germany) were propagated in HeLa cells as described previously (83). The C. trachomatis serovar D strain IC Cal 8 was obtained from the Institute of Ophthalmology, London, United Kingdom. The nonavian C. psittaci BSL 2 strain DC15 (29) and Chlamydia abortus BSL 2 strain S26/3 (DC59) (84) were propagated in BGM cells. All used chlamydial stocks were stored in sucrose-phosphate-glutamic acid buffer at −80°C. IFUs were determined via flow cytometry as previously described (85). Unless indicated otherwise, cells were infected with chlamydia at an MOI of 3 to 5.

### Flow cytometry, microscopy, and colorimetry.

Flow cytometry was performed as previously described (81). Briefly, the cells were carefully detached via a mild trypsin treatment and analyzed on a MACSQuant analyzer using the MacsQuantity software (Miltenyi Biotec). Viability was assessed via trypan blue staining. For the intracellular chlamydial staining and titer determination, the cells were fixed with 2% PFA, permeabilized in PBS/0.5% saponin/0.5% BSA at room temperature (RT) for 30 min, and immunostained with an anti-chlamydial antibody (Thermo Fisher Scientific). Immunofluorescence microscopy was also performed as previously described (81). Prior to permeabilization and antibody staining, cells grown on coverslips were fixed at RT for 15 min in 2% PFA and quenched with 10 mM glycine for 10 min (alternatively, the cells were fixed with 80% acetone in the cold for 15 min). A red fluorescent phalloidin-Alexa fluor 568 conjugate (0.15 μM) was used for the F-actin staining of the fixed cells. All images were captured using an Axiovert 200M/Apotome microscope and analyzed using Axiovision software (Zeiss).

### Live cell imaging.

1×10^5^ HEK293 cells were seeded in μ-dishes (ibidi, 35 mm, low) and infected with C. trachomatis LGV*2*/434/Bu/pGFP::SW2 (kindly provided by Ian N. Clarke, University of Southampton) (27). During the LCI experiments, the culture medium was supplemented with 20 mM HEPES in the absence or presence of heparin. Time-lapse image series were acquired using a Leica THUNDER imaging system (microscope: Leica DMi8; objective: HC PL FLUOTAR L 20×/0.40 DRY; camera: Leica K5) at 37°C with a heating chamber (HT200, ibidi). Time-lapse imaging was started at 24 hpi and continued up to 48 hpi, during which an image series was acquired every 15 min. To reduce the mobility of the examined cells during image acquisition, they were overlaid at 20 hpi with 0.6% low-melting-point agarose in cell culture medium, supplemented with 20 mM HEPES, as indicated above. The recorded LCI movies are available upon request.

### Cell sorting.

For the FACS, which physically separates live cells, cocultivated and mixed RFP- (donor) and β-tub-GFP-HEK293 (acceptor) transfectants (3×10^6^) were examined using a BD FACSAria Cell Sorting System with BD FACSDiva Software (BD Biosciences). For the dual-color cell-sorting approach, the three expected cell types were classified by the presence of one (or both) of the two fluorescent proteins. Gated cells (sorting gate red, 573 nm and sorting gate green, 510 nm) were then separated based on the high fluorescence intensities of the single and double-positive HEK293 transfectants. After cell sorting, the cells were collected, counted, and then immediately lysed in detergent containing lysis buffer.

### Statistical analysis.

Analyses of the obtained data are shown as the means ± standard deviations (SDs) of three or more individual experiments and were calculated using GraphPad Prism 7 (GraphPad Software). Data were analyzed via *t* tests and one-way analyses of variance (ANOVAs) followed by Dunnett’s and/or Tukey’s *post hoc* tests (n.s., not significant; *, *P* < 0.05; **, *P* < 0.01; and ***, *P* < 0.001).

### Data availability.

All data generated or analyzed during this study are included in this published article.
