# Supplementary material for: Chlamydia trachomatis Cell-to-Cell Spread through Tunneling Nanotubes
Source: Microbiol Spectr. 2022 Oct 11;10(6):e02817-22. doi: 10.1128/spectrum.02817-22 (PMC9769577; doi:10.1128/spectrum.02817-22)
Supplement: Supplemental file 1 — Fig. S1 to S8. Download spectrum.02817-22-s0001.pdf, PDF file, 5.9 MB [file spectrum.02817-22-s0001.pdf]

C. trachomatis cell-to-cell spread through tunneling nanotubes (TNTs)

Rico Jahnke[1], Svea Matthiesen[1], Luca M. Zaeck[2], Stefan Finke[2] and Michael R. Knittler[1*]

[1]Institute of Immunology,
Friedrich-Loeffler-Institut,
Federal Research Institute of Animal Health,
Greifswald, Germany

[2]Institute of Molecular Virology and Cell Biology,
Friedrich-Loeffler-Institut,
Federal Research Institute of Animal Health,
Greifswald, Germany

Supplementary Figures & References

Suppl. Fig. 1

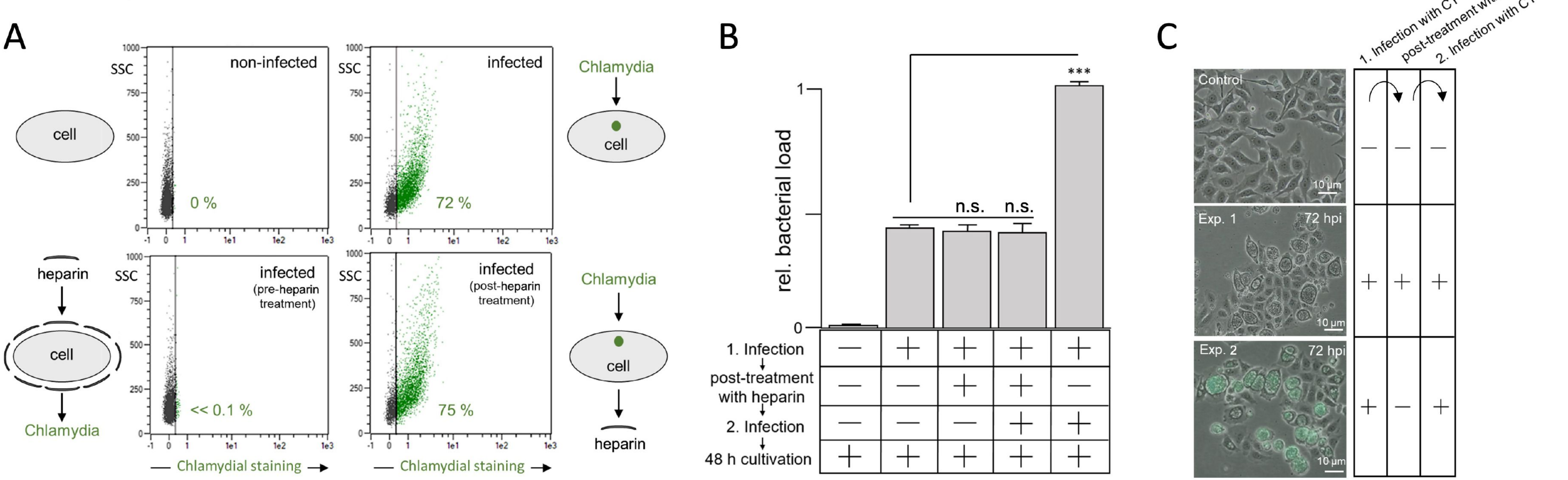

Effects of pre- and post-treatment with heparin on the infection of cells with extracellular chlamydia. A) Flow cytometry of HEK293 cells infected with chlamydia (48 hpi, MOI 5), pre- and post-treated, or not with heparin (5 µg/ml). To detect/quantify chlamydia-positive HEK293 cells (green), the negative cell population (black) was identified and gated via corresponding non-infected controls and then subtracted from the total cell population. **Result:** The findings of these experiments revealed that the addition of heparin before infection almost completely blocks bacterial uptake/infection of HEK293 cells. In contrast, post-treatment with heparin does not affect chlamydial growth in already infected host cells. **B)** PCR analysis of chlamydia-infected HEK293 cells treated or not with heparin between two successive infections. Cells were first infected with *C. trachomatis* (MOI 5) for 2 h. In further optional steps, after washing the cells, a second chlamydial infection (MOI 5) was performed or not in the presence and/or absence of heparin. After 48 hpi the bacterial load was determined by qPCR in which a 151-bp PCR fragment of the singular chromosomal gyrA gene (GenBank accession number:JN795372.1) was amplified with the following gyrA primers: forward, gyrA_for (5′-GCACGTAGAGGAGAACTCGG-3′ [positions 738 to 872]), and reverse, gyrA_rev (5′-GTCAGACTCATCGCGGACAT-3′ [positions 891 to 872]). The analysis was carried out in triplicate in a Bio-Rad CFX96 real-time system (Bio-Rad) (10 s at 95°C, 30 s at 50°C, 44 cycles, followed by a melting-curve analysis: 52°C to 95°C with a temperature increase of 0.5°C). The maximum ΔΔCT value as proxy for bacterial load was set to 1 (n.s., ***, $p < 0.001$, versus infected control ; $n = 3$; mean ± SD). **Result:** The obtained outcome demonstrated that heparin post-treatment of chlamydia-infected cells (1. Infection) efficiently prevents an increase in cellular bacterial load by subsequently added extracellular chlamydia (2. Infection), whereas in the absence of heparin the measured bacterial load of the initial and subsequent infections add up in the infected host cells. Thus, heparin post-treatment of already chlamydia-infected cells prevents subsequent infections with extracellular bacteria present in the medium. **C)** Microscopic analysis of chlamydia-infected HEK293 cells treated or not with heparin between two consecutive chlamydial infections. The cells were first infected with *C. trachomatis* (CT) (MOI 5) for 2 h. After washing, a second infection (MOI 5) with fluorescent *C. trachomatis* (*C. trachomatis* LGV2/434/Bu/pGFP::SW2) (CT-GFP) was performed in the presence (Exp. 1) and/or absence (Exp. 2) of heparin. After 72 hpi, cells were analyzed by using a Nikon Eclipse TS100 fluorescence microscope. **Result:** In line with the two other experiments the microscopic analysis revealed that *C. trachomatis*-infected cells post-treated with heparin do not allow a secondary infection with extracellular GFP-expressing chlamydia. Taken together, this provides ideal experimental conditions for the analysis of direct cell-to-cell transmission of chlamydia without the interfering influence of free bacteria in the medium.

Suppl. Fig. 2

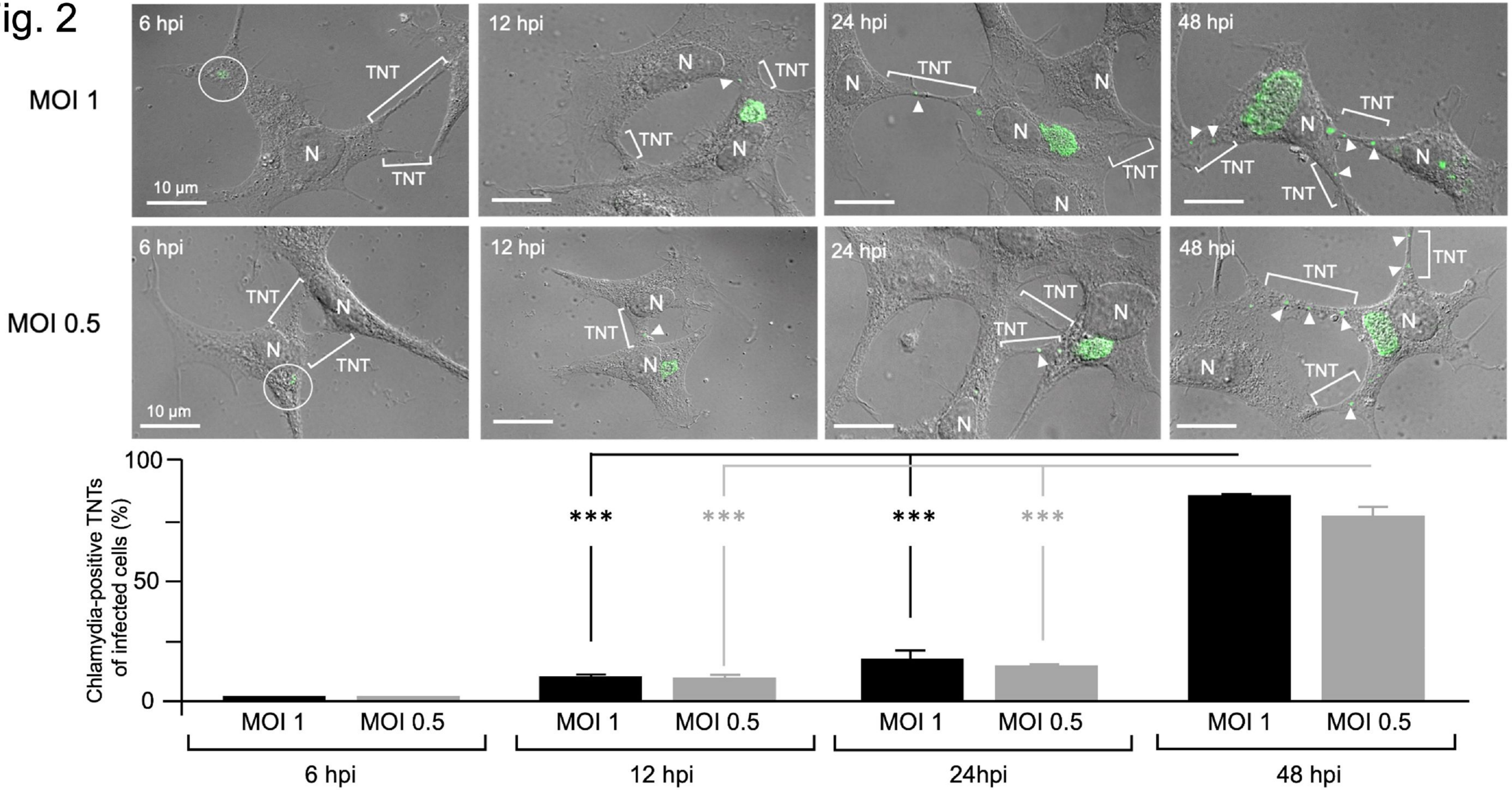

Time-dependent appearance TNT-localized chlamydial structures at different MOIs. Immunofluorescence analysis of *C. trachomatis* (MOI 0.5 and 1) in infected HEK293 cells (6, 12, 24 and 48 hpi) cultivated in the presence of heparin. Cells were fixated with 2% PFA and incubated with anti-chlamydial antibody in the presence of membrane-permeabilizing saponin (0.1%). For indirect immunofluorescence detection an Alexa-488-labeled second antibody was used. Green fluorescence and corresponding phase-contrast images were taken and overlaid using an Axiovert 200M/Apotome microscope (Zeiss) and ImageJ (upper panel). Intracellular chlamydial structures are marked by circle at 6 hpi. Nuclei (N), INCs, and TNTs are indicated. For each MOI, 3 x 100 cells (6, 12, 24 and 48 hpi) connected by multiple membrane conduits were evaluated for the respective number (%) of chlamydia-positive TNTs of infected cells. The respective histogram plot is depicted in the lower panel (***, $p<0.001$, versus 48 hpi samples (MOI 1 and 0.5); $n = 3$; mean ± SD). **Result:** Bacterial structures became increasingly visible in the membrane conduits between 12 and 48 hpi, but not at 6 hpi. Their pronounced appearance in the TNTs of infected cells occurred mainly at 48 hpi.

Suppl. Fig. 3

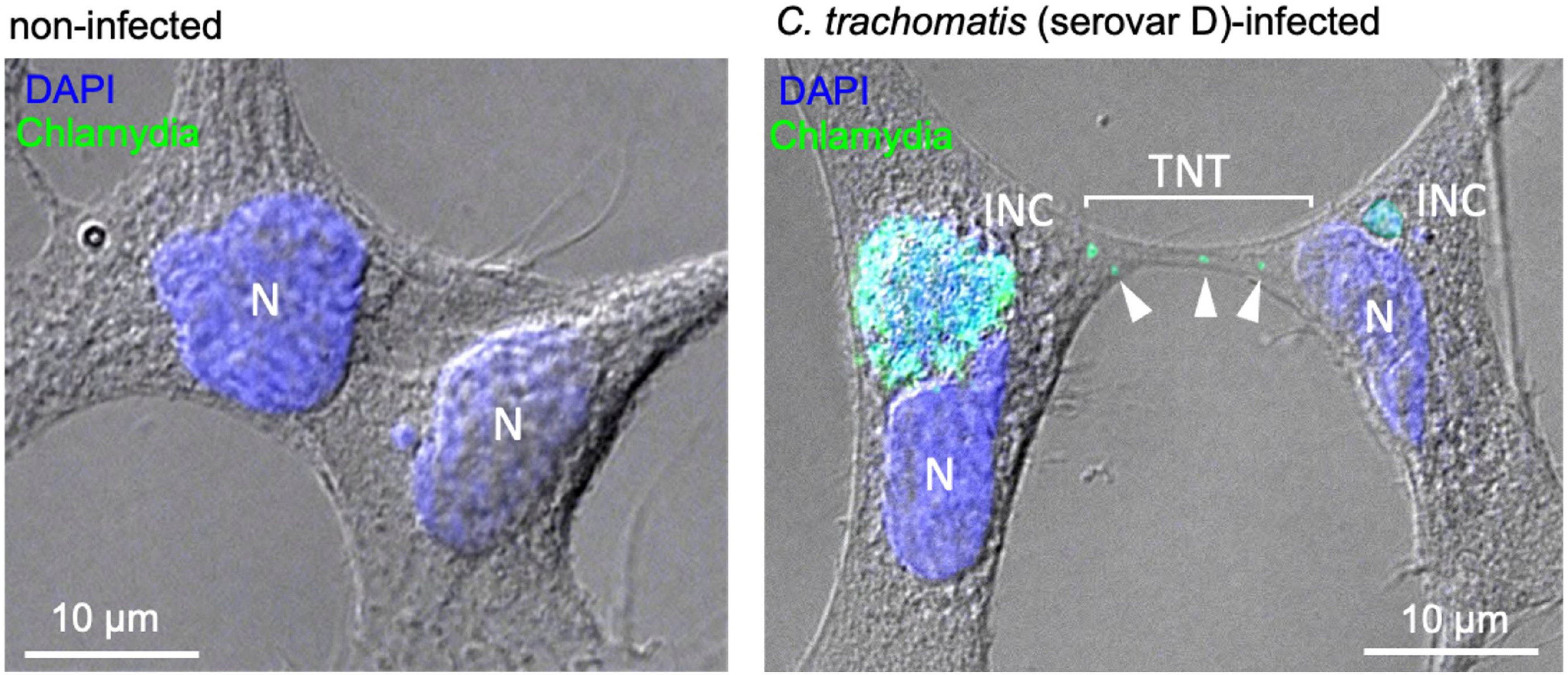

Presence of chlamydia within the cytoplasm and interconnecting TNTs of *C. tracho*matis serovar D-infected HEK293 cells. Immunofluorescence analysis of chlamydia (green) (48 hpi) in infected PFA-fixed HEK293 cells (MOI 3). DNA was stained via DAPI (blue). Fluorescence and corresponding phase-contrast images were taken and overlaid to visualize the presence of interconnecting TNTs. White arrowheads indicate chlamydial structures found in the context of TNTs. The overlay image on the left shows the corresponding control experiment with non-infected HEK293 cells. TNTs, INCs, and nuclei (N) are indicated. **Result:** Bacterial structures are present not only in the cell body of *C. tracho*matis serovar D-infected HEK293 cells but also within TNTs between interconnected cell partners.

Suppl. Fig. 4

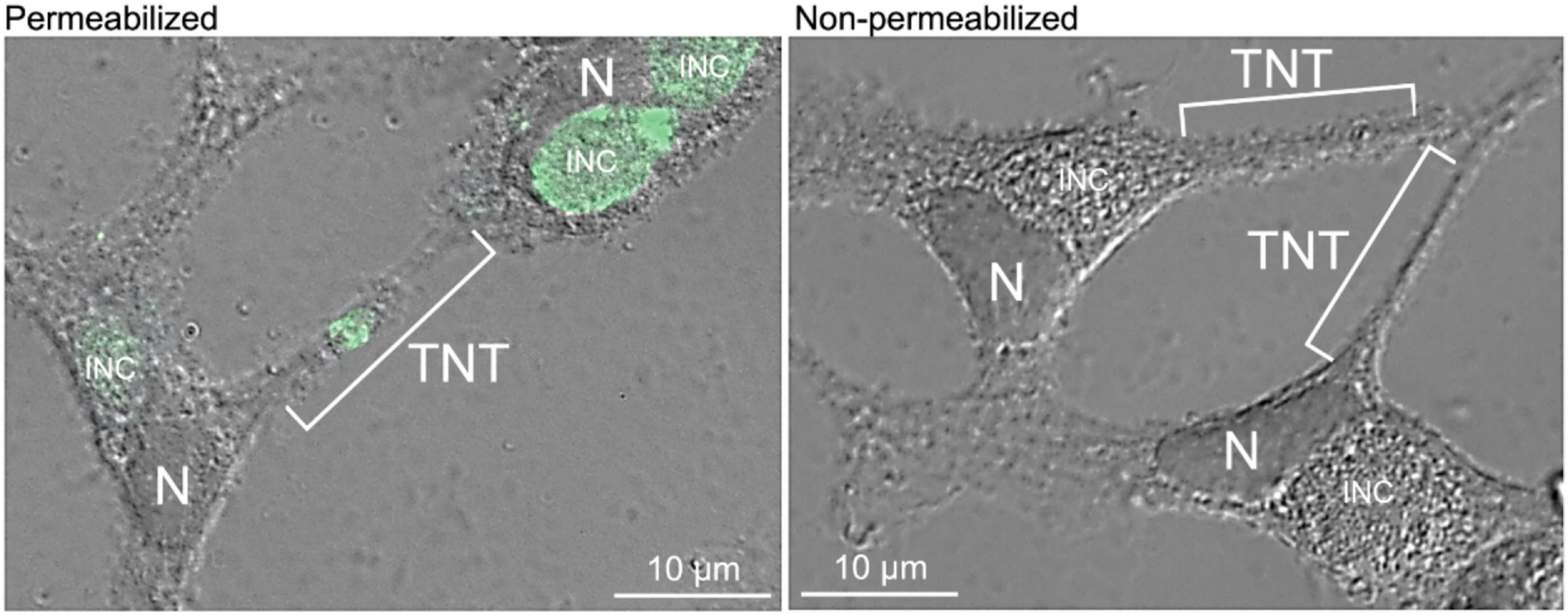

TNT-localized chlamydial structures are immunodetectable exclusively after cell permeabilization. Immunofluorescence analysis of *C. trachomatis* (MOI 3) in HEK293 cells cultivated in the presence of heparin (48 hpi). Cells were fixated with 2% PFA and incubated with anti-chlamydial antibody in the presence of membrane-permeabilizing saponin (0.1%) (left panel, permeabilized). In addition, cells without permeabilization were first incubated with an anti-chlamydial antibody and than fixated with 2% PFA (right panel, non-permeabilized). For indirect immunofluorescence detection an Alexa-488-labeled second antibody was used. Green fluorescence and corresponding phase-contrast images were taken and overlaid using an Axiovert 200M/Apotome microscope (Zeiss). Nuclei (N), INCs, and TNTs are indicated. **Result:** Detection of chlamydia within TNTs is possible only after permeabilization of the infected cells, indicating that the observed bacterial structures are located exclusively inside the interconnecting membrane conduits.

Suppl. Fig. 5

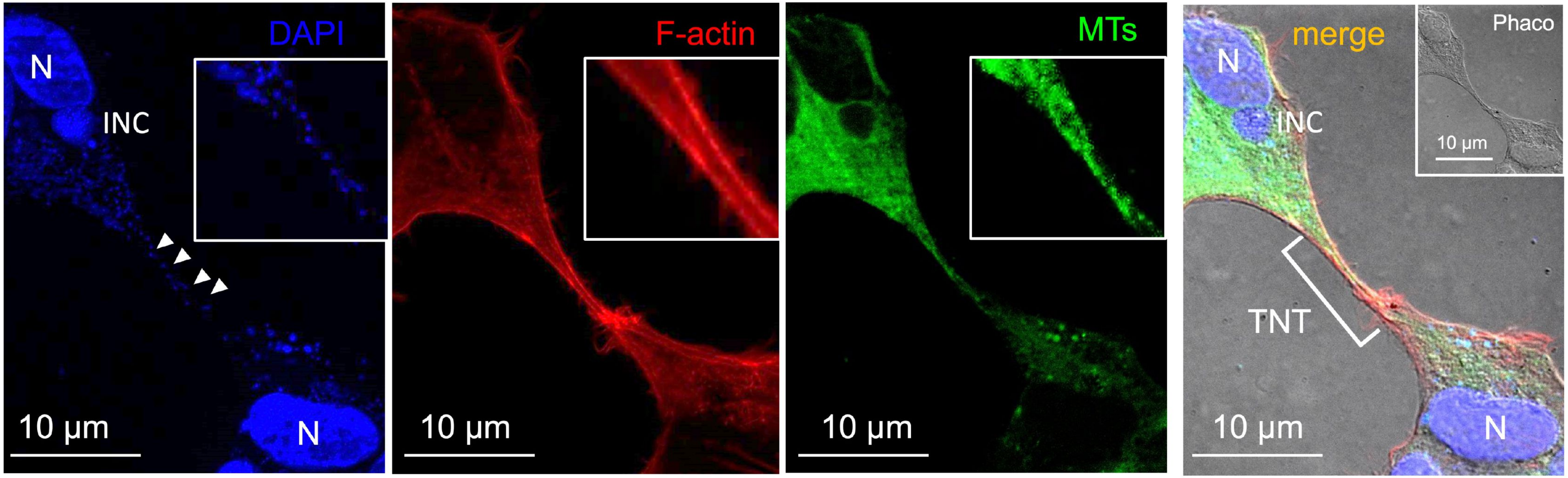

Chlamydia containing TNTs are characterized by different cytoskeleton components. The depicted figure shows an fluorescence analysis of chlamydia-infected HEK293 cells (48 hpi, MOI 3) in the presence of heparin. Cells were rapidly fixed with 2% PFA in the cold and then stained with DAPI (nucleus and chlamydia, blue) and phalloidin-Alexa Fluor 568 conjugate (0.15 µM, F-actin, red). Fluorescence-labeled β-tubulin is visible in green (MTs). Images were captured using an Axiovert 200M/Apotome microscope (Zeiss). The insets within the three fluorescence images (DAPI, F-actin, and MTs) show a selected section of the TNT. Nuclei (N), INC, chlamydia (white arrowheads) and TNT are indicated. **Result:** The analysis revealed that in addition to MTs, F-actin was also found to be a structural component of chlamydia-containing TNTs between interconnected infected HEK293 cells suggesting that distinct cytoskeleton fibers stabilize the formation of TNTs. In particular, F-actin appears to be highly concentrated at the junction of TNT-connected cells.

Suppl. Fig. 6

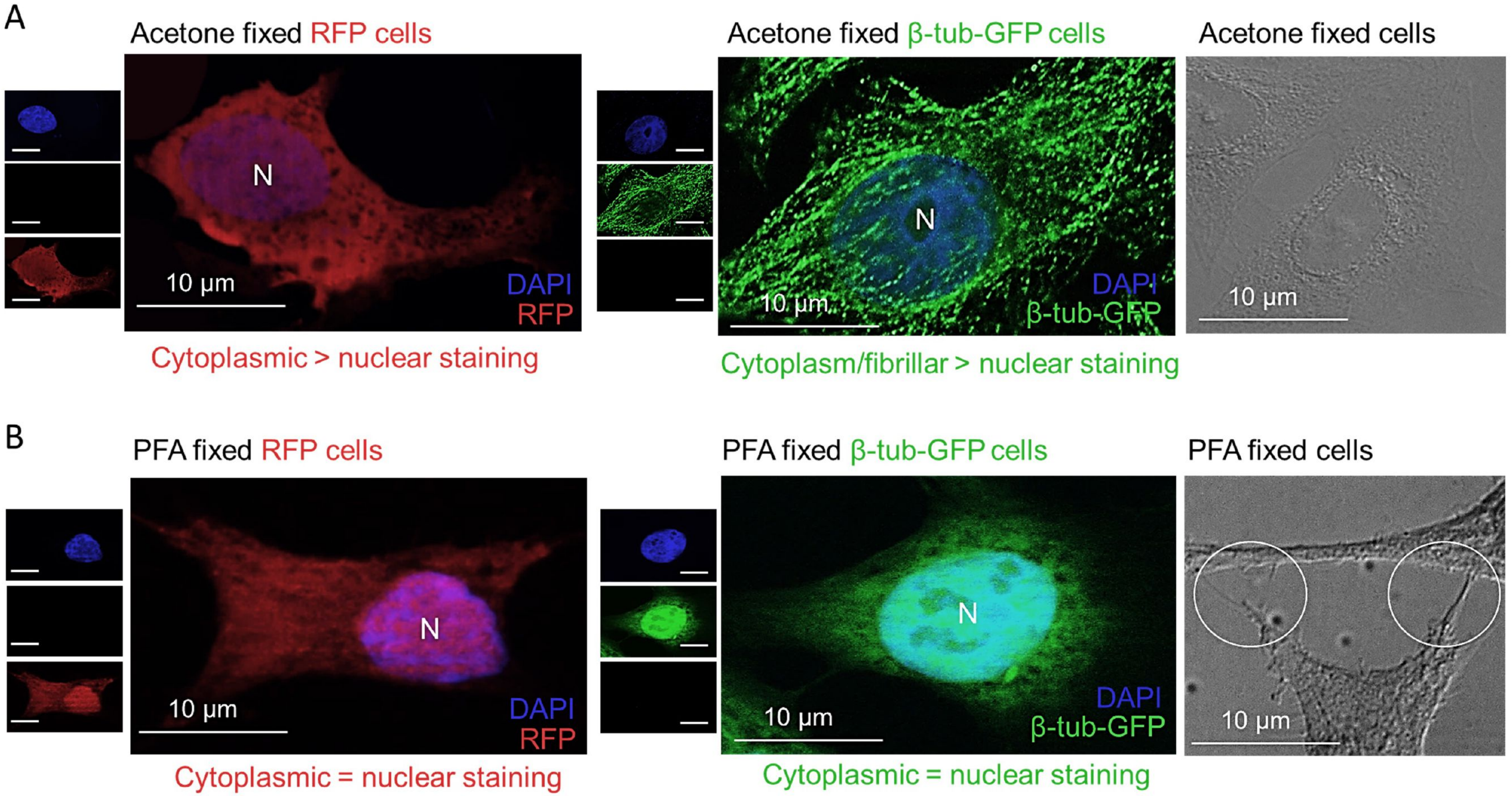

Establishment of donor/acceptor cell lines expressing fluorescent protein markers with freely diffusible or filamentous properties. HEK293 transfectants stably expressing RFP (red) or β-tub-GFP (green) were fixed with 80% acetone **(A)** or 2% PFA **(B)** and stained for DNA via DAPI (blue) (left and middle panels). The visibility of TNTs was checked by taking phase-contrast images of the differently fixed HEK293 cells (right panel). **Result:** The analysis of acetone-fixed preparations revealed the expected microscopic features of RFP and β-tub-GFP expressing HEK293 transfectants. Soluble RFP is characterized by a cytoplasmic distribution (cytosol and nucleus (N)), whereas a cell-filling MT network was observed for β-tub-GFP. However, with acetone as a fixative, the membrane structures/boundaries of cells and TNTs were only faintly visible, if at all. In the case of PFA-fixation, RFP and β-tub-GFP showed an additional characteristic nuclear staining, as described previously (suppl. references 1, 2, and 3) and all TNT structures were clearly detectable. No spillover of green/red fluorescence was observed for the RFP- or β-tub-GFP-expressing HEK293 transfectants with either fixation method. Since the TNT structures were clearly visible when PFA was used as a fixative, this cell preparation method was used for all further immunofluorescence experiments.

Suppl. Fig. 7

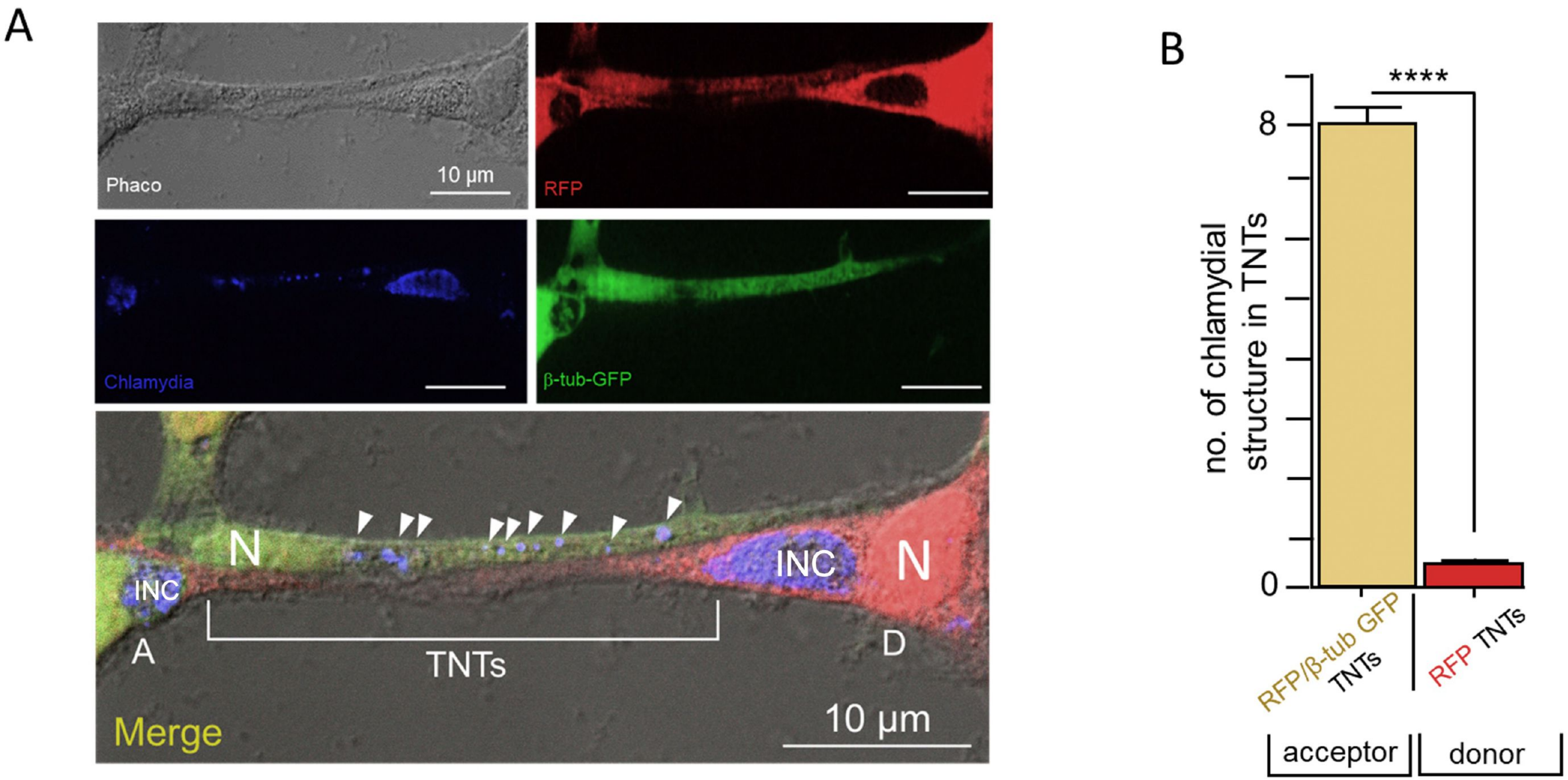

Chlamydia specifically uses the TNT formed by the HEK293 acceptor cells. A) Immunofluorescence analysis of chlamydia (blue, Alexa 405-coupled sec. antibody) in infected RFP-donor HEK293 cells (24 hpi, MOI 3) co-cultured with non-infected β-tub-GFP acceptor HEK293 cells (48 hpi) in the presence of heparin. Fluorescence and corresponding phase-contrast images were taken and then overlaid. Donor (D), acceptor (A), INC, nucleus (N), chlamydial structures (white arrowheads), and TNTs are indicated. **B)** For quantitative analysis, 35 antiparallel TNT structures connecting donor and acceptor HEK293 cells were analyzed for the number of chlamydial structures. The obtained results are shown as a histogram (****, $p < 0.0001$, acceptor versus donor HEK293 cells; $n = 35$; mean ± SD). **Result:** For almost all antiparallel TNT pairs analyzed, transferred chlamydial structures were found exclusively in TNTs formed by β-tub-GFP-acceptor cells, suggesting that chlamydia specifically uses the MT motor proteins (e.g., dynein) of acceptor cells to be transported in their direction.

Suppl. Fig. 8

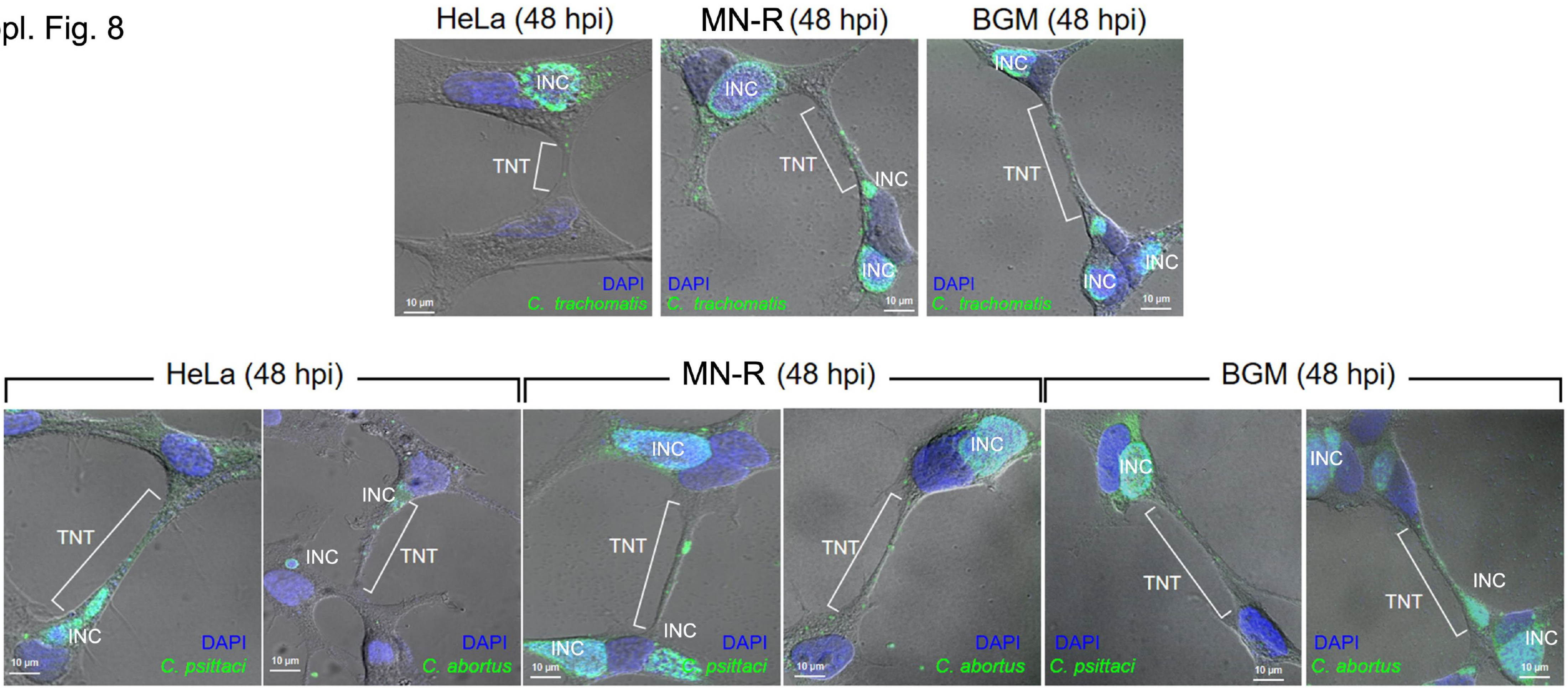

Detection of *C. trachomatis*, *C. psittaci* and *C. abortus* in TNTs of different infected cell lines. Immunofluorescence analysis of chlamydia *(C. trachomatis, C. psittaci und C. abortus,* green) in infected 2% PFA-fixed HEK293, MN-R, and BGM cells (MOI 3) cultivated in the presence of heparin (48 hpi). DNA was stained via DAPI (blue). Fluorescence and corresponding phase-contrast images were taken and overlaid to visualize the presence of interconnecting TNTs (indicated by white brackets). INCs are indicated. **Result:** The microscopic results obtained show that TNT-mediated bacterial transfer is indeed a conserved strategy across different chlamydia strains and infected cell types.

Supplementary References

1. Tsuzuki Y, Sanami S, Sugimoto K, Fujita S. 2021. Pseudo-nuclear staining of cells by deep learning improves the accuracy of automated cell counting in a label-free cellular population. J Biosci Bioeng 131: 213-218

2. Akoumianaki T, Kardassis D, Polioudaki H, Georgatos SD, Theodoropoulos PA. 2009. Nucleocytoplasmic shuttling of soluble tubulin in mammalian cells. J Cell Sci 122: 1111-1118

3. Schwarzerova K, Bellinvia E, Martinek J, Sikorova L, Dostal V, Libusova L, Bokvaj P, Fischer L, Schmit AC, Nick P. 2019. Tubulin is actively exported from the nucleus through the Exportin1/CRM1 pathway. Sci Rep 9: 5725
